# Comparison of alfalfa plants overexpressing glutamine synthetase with those overexpressing sucrose phosphate synthase demonstrates a signaling mechanism integrating carbon and nitrogen metabolism between the leaves and nodules

**DOI:** 10.1002/pld3.115

**Published:** 2019-01-31

**Authors:** Harmanpreet Kaur, Amanda Peel, Karen Acosta, Sayed Gebril, Jose Luis Ortega, Champa Sengupta‐Gopalan

**Affiliations:** ^1^ Department of Plant and Environmental Sciences New Mexico State University Las Cruces New Mexico; ^2^ Department of Learning, Teaching and Curriculum University of Missouri Columbia Missouri; ^3^ Department of Biochemistry and Biophysics Perelman School of Medicine University of Pennsylvania Philadelphia Pennsylvania; ^4^ Department of Horticulture Sohag University Sohag Egypt

**Keywords:** forage quality, malate dehydrogenase, nitrogenase activity, phosphoenolpyruvate carboxylase, SPS and GS activity, sucrose synthase

## Abstract

Alfalfa, like other legumes, establishes a symbiotic relationship with the soil bacteria, *Sinorhizobium meliloti*, which results in the formation of the root nodules. Nodules contain the bacteria enclosed in a membrane‐bound vesicle, the symbiosome where it fixes atmospheric N_2_ and converts it into ammonia using the bacterial enzyme, nitrogenase. The ammonia released into the cytoplasm from the symbiosome is assimilated into glutamine (Gln) using carbon skeletons produced by the metabolism of sucrose (Suc), which is imported into the nodules from the leaves. The key enzyme involved in the synthesis of Suc in the leaves is sucrose phosphate synthase (SPS) and glutamine synthetase (GS) is the enzyme with a role in ammonia assimilation in the root nodules. Alfalfa plants, overexpressing *SPS* or *GS*, or both showed increased growth and an increase in nodule function. The endogenous genes for the key enzymes in C/N metabolism showed increased expression in the nodules of both sets of transformants. Furthermore, the endogenous *SPS* and *GS* genes were also induced in the leaves and nodules of the transformants, irrespective of the transgene, suggesting that the two classes of plants share a common signaling pathway regulating C/N metabolism in the nodules. This study reaffirms the utility of the nodulated legume plant to study C/N interaction and the cross talk between the source and sink for C and N.

## 1 INTRODUCTION

Carbon (C) and nitrogen (N) assimilations are closely interconnected and plant growth is dependent on this interaction. Plants possess intricate regulatory machineries that coordinate N assimilation with C metabolism and meet the demands created by plant growth and development (Ljung, Nemhauser, & Prata, 2015). The control of the C–N interaction requires a complex network involving signals emanating from both C and N metabolisms (Amiour et al., 2012; Coruzzi & Zhou, 2001; Miller, Fan, Shen, & Smith, 2008; Sang, Sun, & Yang, 2012). These metabolic signals interact with hormones that respond to the N supply and regulate metabolism and development (Argueso, Ferreira, & Kieber, 2009; Coruzzi & Zhou, 2001; Ljung et al., 2015; Sakakibara, Takei, & Hirose, 2006).

Photosynthesis drives C and N assimilation by providing assimilatory power (reduced ferredoxin, NADPH, ATP) and the C skeletons required for the assimilation of N. With regard to the origin of C skeletons required for glutamate/glutamine (Glu/Gln) synthesis in illuminated leaves, the 2‐oxoglutarate molecules could be newly synthesized via photosynthesis or derived from metabolites in the night. Based on isotopic tracing experiments on illuminated leaves of *Brassica napus*, Gauthier et al. (2010) showed that night stored carbon, such as organic acids, played a significant role in nitrogen assimilation and Glu synthesis. Similar study by Abadie, Lothier, Boex‐Fontvieille, Carroll, and Tcherkez (2017), using NMR‐based metabolic flux analysis in illuminated leaves of Helianthus annuus, has shown that the C for Glu/Gln synthesis depends on previous C stored in the vacuoles and that it is citrate and other organic acids and not sucrose (Suc). However, such experiments have not been done for heterotrophic tissues.

Sucrose is the main photoassimilate that is exported from the leaves to supply the rest of the plant with reducing power and C skeletons needed for growth and for the synthesis of storage reserves (Lunn & Furbank, 1999), thus playing a role in both metabolism and development (Eveland & Jackson, 2012; Lunn & MacRae, 2003). Suc also acts as a signaling molecule for regulation of gene expression (Loreti, Bellis, Alp, & Perata, 2001; Ruan, 2012; Smeekens, 2000; Smeekens & Hellmann, 2014; Tognetti, Pontis, & Martinez‐Noel, 2013; Weise, Elzinga, Wobbes, & Smeekens, 2004; Wind, Smeekens, & Hanson, 2010).

Because large amounts of N are invested in the photosynthetic machinery, optimal CO_2_ assimilation through photosynthesis requires an adequate N supply. Plants acquire N from two principal sources: (a) the soil, as nitrate through commercial fertilizer or mineralization of indigenous organic matter and (b) in the case of legumes, the atmosphere, through symbiotic N_2_ fixation. Nitrate is converted into NH_4_^+^ by nitrate reductase (NR) and nitrite reductase (NiR) while atmospheric N_2_ is reduced to NH_4_^+^ by the bacterial enzyme, nitrogenase. Significant amounts of NH_4_^+^ are also derived from photorespiration and phenylpropanoid biosynthesis (Sengupta‐Gopalan & Ortega, 2015). The primary assimilation of ammonia into amino acids involves the ATP‐dependent amination of Glu to Gln by the enzyme glutamine synthetase (GS). This reaction is followed by the reductive transfer of the amide‐amino group of Gln to α‐ketoglutarate (α‐KG) to yield two Glu, with the reaction catalyzed by ferredoxin (Fd^−^) or NADH‐glutamate synthase (GOGAT).

Alfalfa, like all other legumes, establishes a symbiotic relationship with the soil bacteria, *Sinorhizobium meliloti*, which results in the formation of a novel organ, the root nodule. The root nodule contains the bacteria enclosed in a membrane‐bound vesicle, the symbiosome (Clarke, Loughlin, Day, & Smith, 2014), where it fixes atmospheric N and converts it into ammonia using the bacterial enzyme, nitrogenase. The ammonia released into the cytoplasm from the symbiosome is assimilated into Gln using C skeletons produced by the metabolism of Suc, which is imported into the nodules from the leaves. A product of Suc metabolism, malate, moves into the symbiosome and acts as a source of energy for nitrogenase activity (Oldroyd & Downie, 2008). While the key enzyme in Suc synthesis is sucrose phosphate synthase (SPS), the enzyme with the key role in ammonia assimilation is GS.

Sucrose phosphate synthase is regulated by a hierarchy of several interacting mechanisms, which includes transcriptional regulation, covalent modification via reversible phosphorylation (Huber & Huber, 1996; Toroser & Huber, 1997), and allosteric regulation via metabolites, Glc‐6‐P and phosphate (Lunn, Gillespie, & Furbank, 2003). Even though the major role of SPS is in photosynthetic tissues, SPS is also found in heterotrophic tissues like the roots, stem, and nodules (Aleman et al., 2010; Haigler et al., 2007). SPS is encoded by a small gene family. There are two families (A and B) present in the genomes of alfalfa, *Medicago truncatula* and pea (Aleman et al., 2010). While *SPSB* exhibits leaf‐specific expression, *SPSA* is expressed in the N_2_‐fixing zone of the nodules and in the vasculature of the nodules, roots, stem, and leaves (Aleman et al., 2010; Grimes, 2013). It is not known what the function of SPS is in the nodules but one could envision it having a role along with sucrose synthase (SucS) and invertase, in maintaining optimum levels of Suc to provide substrates for starch and cellulose synthesis, ammonia assimilation, and nitrogenase activity, and for functioning as a signaling molecule for gene expression in the nodules.

Glutamine synthetase is the key enzyme in the conversion of inorganic N to an organic form and there are two major isoforms of GS: cytosolic GS (GS_1_) occurring in the cytosol and chloroplastic GS (GS_2_), the latter, though nuclear encoded, is located in the chloroplasts/plastids. In the leaves, GS_2_ assimilates ammonia from nitrate reduction and reassimilates ammonia released during photorespiration (Oliveira, Brears, Knight, Clark, & Coruzzi, 2002). GS_1_ is the predominant isoform found in non‐photosynthetic tissues and its role is more complex due to its numerous isoforms (Bernard & Habash, 2009; Lea & Miflin, 2011). In roots, GS_1_ assimilates ammonia derived from NO_3_^−^ reduction or from the soil (Bernard & Habash, 2009; Lothier et al., 2010; White, Prell, James, & Poole, 2007), and in the leaves and stem, GS_1_ is located in the vasculature and plays a role in N translocation. In root nodules, the primary function of GS_1_ is the rapid assimilation of ammonia excreted into the plant cytosol by N_2_‐fixing bacteroids (Oldroyd & Downie, 2008). There are two *GS*_*1*_ gene members in alfalfa, although constitutively expressed in all organs, the expression of both isoforms, specifically *GS1a*, is the highest in the nodules. The regulation of *GS*_*1*_ gene expression is not limited to transcription (Sengupta‐Gopalan & Ortega, 2015). It has been shown that *GS*_*1*_ is regulated at the level of transcript stability, mediated by its 3′UTR (Ortega et al., 2006; Simon & Sengupta‐Gopalan, 2010) and at the translational level by the 5′UTR (Ortega, Wilson, & Sengupta‐Gopalan, 2012). Furthermore, GS is subject to extensive posttranslational modification like phosphorylation (Finnemann & Schjoerring, 2000; Lima, Seabra, Melo, Cullimore, & Carvalho, 2006), ubiquitination, and binding with other proteins (Seabra, Silva, & Carvalho, 2013). GS is also subject to regulation at the level of holoenzyme turnover (Ortega, Roche, & Sengupta‐Gopalan, 1999).

Since SPS and GS play key roles in primary metabolism, efforts have been made to modulate the expression of these genes using transgenic approaches. The outcomes of overexpression of *SPS* have been quite varied, but in general, increased SPS activity is associated with the production of new sinks and increased sink strength (Baxter, Foyer, Turner, Rolfe, & Quick, 2003; Gebril et al., 2015; Haigler et al., 2007; Ishimaru et al., 2008; Laporte et al., 2001; Micallef et al., 1995; Nguyen‐Quoc, N'Tchobo, Foyer, & Yelle, 1999; Park, Canam, Kang, Ellis, & Mansfield, 2008; Park, Canam, Kang, Unda, & Mansfield, 2009; Seger, Gebril, Tabilona, Peel, & Sengupta‐Gopalan, 2015). Similarly, there have been several attempts to modulate the levels of GS_1_ enzyme in different plants using genetic engineering tools with the goal of improving the plant performance. However, no overriding picture has emerged from all these studies (Bao et al., 2014; Carvalho, Lopes‐Cardoso, Lima, Melo, & Cullimore, 2003; Fuentes, Allen, Ortiz‐Lopez, & Hernández, 2001; Harrison, de Crescenzo, Sené, & Hirel, 2003; Kirby, Gallardo, Man, & El‐Khatib, 2006; Oliveira et al., 2002; Ortega, Temple, Bagga, Ghoshroy, & Sengupta‐Gopalan, 2004; Seger, Ortega, Bagga, & Sengupta‐Gopalan, 2009; Seger et al., 2015; Temple, Knight, Unkefer, & Sengupta‐Gopalan, 1993; Temple, Bagga, & Sengupta‐Gopalan, 1994, 1998; Thomsen, Eriksson, Møller, & Schjoerring, 2014).

Legumes are unique in that they have nodules which function as a C sink and N source while the leaves function as a N sink and C source, thus making it an ideal system for studying C/N interaction and the cross talk between the source and sink for C and N. To determine if there is any inter/codependence between C and N metabolic pathways, this study was undertaken to compare *GS*_*1*_ overexpressing alfalfa plants (*35S‐GS*) with the *35S‐SPS* transformants. Our hypothesis is that increased SPS expression in the leaves would lead to an increase in Suc transport to the nodules while upregulating GS_1_ would increase the nitrogen assimilation in the nodules. The most intriguing outcome of this study was that both classes of transformants, irrespective of the transgene, showed a very similar response at different levels.

## 2 MATERIALS AND METHODS

### 2.1 Gene manipulations

The *CaMV 35S‐SPS* (*ZmSPS*) and the *CaMV 35S‐GS (Gmglnb*_*1*_) constructs are as described by Seger et al. (2015). Plasmid *pCAMBIA 2300* was used as the control vector for transformation.

### 2.2 Plant transformation and growth conditions

*Agrobacterium*‐mediated plant transformations were carried out as described by Gebril et al. (2015). DNA was isolated from the leaves of alfalfa using a DNeasy Mini Plant Kit (Qiagen). The DNA was subjected to PCR using the *NPTII* primer set. The randomly selected PCR‐positive transformants from tissue culture, three with *Cambia 2300* (control), three with *35S‐SPS* and three with *35S‐GS* gene constructs, were transferred into the greenhouse. Plants were inoculated with *S. meliloti* 2011 to initiate symbiotic N_2_ fixation, and then fed N‐free Hoagland's nutrient solution, weekly. Since alfalfa is self‐incompatible and seeds cannot be obtained by selfing, plants were clonally propagated to make biological replicates. This was done by cutting the stem with apical meristem, dipping the cut end in a rooting hormone, then placing them in wet vermiculite in pots. Cuttings were also inoculated after acclimation and fed N‐free Hoagland, weekly. For each control plant and individual transformants, three to five clones were analyzed as biological replicates and averaged or pooled for experiments. The plants were grown in the greenhouse with full sunlight during the day along with supplemental LED grow lights (LIFTED, Rio Rancho, NM) for an extended light period during the winter.

### 2.3 RNA isolation and RT‐PCR

Total RNA was isolated from alfalfa tissues by the LiCl precipitation method (Ortega et al., 2006). A quantity of 2 μg of DNase‐treated RNA was used to prepare cDNA using a Superscript III first strand synthesis RT‐PCR kit (Invitrogen). The standard synthesis profile and reaction conditions were used as described in the Invitrogen protocol.

To perform semiquantitative RT‐PCR, the cDNA for each sample was subjected to PCR amplification using the primer sets for the different genes: *ZmSPS1*,* Gmglnb1*, and endogenous genes *SPSA, SPSB, GS1a, GS1b*, and *Actin* (Table 1). The primers were designed using IDT primer quest tool software and PCR was carried out using a OneTaq Hot Start PCR Kit (New England Biolabs). The amount of cDNA used and the number of cycles in the thermocycler were varied depending on the expression level of the endogenous genes in the leaves and nodules of alfalfa plants. The number of cycles was fine tuned to ensure that the reaction was terminated during the linear phase of amplification. *Actin* primers were used as an internal control. The products, following PCR, were subjected to electrophoresis and the band intensity for all the amplicons was measured and the values were adjusted based on the band intensity of the amplicon for *Actin*.

**Table 1:** Primers used to monitor the expression of the *GS* and *SPS* genes in the alfalfa transformants

Gene	Primer direction	Sequence
*Medicago sativa Actin*	Forward	5′‐ACTTAACCCAAAGGCCAATAGA‐3′
	Reverse	5′‐TGCTCATACGGTCAGCAATAC‐3′
*M. sativa SPSA*	Forward	5′‐CCACTCACTTGGTCGAGATAAG‐3′
	Reverse	5′‐TATGCCACTTGCCCATACAG‐3′
*M. sativa SPSB5*	Forward	5′‐CTCTTCACGCGCCAAATATCATCTC‐3′
	Reverse	5′‐AGCTCTTCCGCTTCTATCCTTCTC‐3′
*M. sativa GS1a*	Forward	5′‐CACAAATCAAGCTCCAGGACAAGATAG‐3′
	Reverse	5′‐CATCTCCAGCAGAAATACCAACAGAAG‐3′
*M. sativa GS1b*	Forward	5′‐TGGTCCCTCAGTTGGTATCT‐3′
	Reverse	5′‐TGTGTGTGTGGTGGCTTATG‐3′
*Zea mays SPS1*	Forward	5′‐CCCGAAGAAGAACATCACTACC‐3′
	Reverse	5′‐CGTCGATGTCATCTCTGTTACC‐3′

### 2.4 Protein isolation and analysis

Leaf and nodule tissues from biological replicates (three to five clones) were harvested for each independent transformant, and three independent transformants for each class were immediately placed in liquid N, and stored at −80°C until experiments were done. Tissues were ground in liquid N and homogenized with 6 volumes of extraction buffer. The SPS extraction buffer consisted of 50 mM HEPES, pH 7.5, 20% (v/v) glycerol, 5% (v/v) ethylene glycol, 5 mM EDTA, 5 mM magnesium acetate, 0.5% Triton X‐100, 10 mM DTT, and a protease inhibitors cocktail (Thermo Fisher). The GS extraction buffer consisted of 50 mM Tris‐HCl pH 8.0, 20% glycerol (v/v), 5% ethylene glycol (v/v), 1 mM MgCl_2_, 1 mM DTT, 1 mM EDTA, and a protease inhibitors cocktail. For SPS activity assays, the total protein extract was desalted in Sephadex G25 columns with desalting buffer (25 mM HEPES pH 7.5, 2.5 mM magnesium acetate, 20% glycerol (v/v), 5% ethylene glycol (v/v), and 1 mM EDTA). Protein concentration was measured using the Bradford protein assay (BioRad, Hercules, CA) with bovine serum albumin for protein standard concentrations.

### 2.5 Enzyme activity

SPS activity was assayed by quantifying the fructosyl moiety of Suc using the anthrone test (Seger et al., 2015) and the activity is expressed as nmol Suc‐P mg^−1 ^protein min^−1^. Total GS activity was measured using the transferase assay as described by Seger et al. (2015). Transferase units were calculated from a standard curve of γ‐glutamyl hydroxamate. The activity is reported as μmole of γ‐glutamyl hydroxamate produced per mg of protein at 30°C.

### 2.6 Western blotting

The protein extracts used for enzyme activities were subjected to SDS–PAGE followed by western blotting. The percentage of these gels varied from 7% to 12% depending on molecular weight of all different proteins. The amount of protein loaded on the gels for each enzyme was adjusted to ensure that the bands were not saturated. The fractionated protein from these gels was electroblotted on the Immobilon‐P, PVDF membrane (Milipore, Bedford, MA). The detection of polypeptides was performed using polyclonal antibodies raised against soybean GS_1_ (Ortega et al., 2006), SPS (Agrisera, Sweden), SucS (provided by Dr. Raymond Chollet, University of Nebraska, Lincoln), PEPC, AS, NADH‐GOGAT, and nodule‐enhanced malate dehydrogenase (neMDH) (provided by Dr. Carol Vance, University of Minnesota, MN). The immunoreactive bands were visualized with alkaline phosphatase linked secondary antibody using nitroblue tetrazolium (NBT) and 5‐bromo‐4‐chloro‐3‐indoyl‐phosphate (BCIP) as substrates. The immunoreactive bands were quantified using the KODAK 1D image analysis system (CARESTREAM). Experiments were performed at least three to four times and only representative results were presented.

### 2.7 Carbohydrate analysis

Carbohydrate analysis was done using the anthrone method essentially as described in our earlier papers (Aleman et al., 2010; Gebril et al., 2015). For Suc and starch analysis, plants (35–40 days post‐inoculation) were kept under supplemental light for 48 hr, and leaves and nodules were harvested at 3:00 p.m.

### 2.8 Leaf gas exchange measurement

Gas exchange or net photosynthetic rates (*P*_net_) was measured with a Li‐Cor 6400‐05 conifer chamber with an external light source attached to an infrared gas analyzer‐based photosynthesis system (Li‐Cor Inc., Lincoln, Nebraska). Measurements were performed on mature trifoliate leaves between 10:00 a.m. and 12:00 p.m. after plants had been under supplemental light for 2 hours. Net photosynthetic rates were measured at a CO_2_ flow rate of 400 μmol/s and an internal CO_2_ of 400 μmol CO_2 _mol^−1^. Trifoliate leaf area was measured using a Li‐Cor LI3000 portable area meter (Li‐Cor Inc., Lincoln, Nebraska). Two measurements were done on each plant, consisting of three biological replicates for each of the three independent transformants and the average of the three values for each transformant was calculated and presented.

### 2.9 Nitrogenase activity

Nitrogenase activity of nodules was measured using an acetylene reduction assay (ARA) method described by Hardy et al. (1968). Alfalfa plants were removed from pots and excess vermiculite was shaken off the roots. These roots were placed in mason jars having Teflon lined septa on the top. The reaction was started by replacing 10% of internal air with acetylene (C_2_H_2_), which was generated from calcium carbide (CaC_2_) and water. The contents in the jar were shaken after every half an hour and 3 ml of acetylene/ethylene mixture was taken out at 0, 30, 60, and 90 min using a syringe. The mixture was kept in vacuum tubes until analysis. Empty jars with 10% acetylene was used as a blank to determine any background level. The amount of ethylene production was measured using gas chromatography (GC). Using a tight syringe, 1.0 ml from each vacuum tube containing acetylene/ethylene mixture was taken and injected into a GC. A standard curve with known concentrations of pure ethylene was generated and nitrogenase activity was measured as nmol ethylene per plant.

### 2.10 Biomass measurements

The primary transformants were moved from tissue culture containers to pots with vermiculite and grown in the greenhouse. The plants were inoculated with *S. meliloti* and grown for 2–3 months to produce the “mother plants.” Cuttings of the same size from these plants were made and planted in pots (vermiculite), and were inoculated. The plants were allowed to grow until the onset of flowering and then cut down and allowed to grow until the onset of flowering. At this time, the aerial parts of the plants were harvested weighed and then put in bags for drying. The fresh and dry biomass were measured. Since the control plants flowered later than the other two classes of transformants, they were allowed to grow for another 20 days before being cut.

### 2.11 Forage quality

Three sets of each control, *35S‐SPS*, and *35S‐GS* alfalfa transformants grown under symbiotic conditions were used to determine forage quality. These plants were treated the same way as for biomass measurement. About 10–12 shoots from each class of plants were harvested, pooled, and fresh samples were sent to SDK laboratories (Hutchinson, KS) for complete N profile and fiber analysis.

### 2.12 Statistical analysis

The data were analyzed with ANOVA comparison test using SAS software (v9.4). Each bar on the graphs is the average of three biological replicates and bars represent the value of standard deviation. Significant differences were evaluated using ANOVA planned contrast and shown by asterisks. Single asterisk (*) indicates 0.01 < *p* < 0.05 and double asterisk (**) indicates 0.001 < *p* < 0.01.

## 3 RESULTS

### 3.1 The *35S‐SPS* and the *35S‐GS* transformants showed the accumulation of the transgene product corresponding to the gene construct in both the leaves and nodules

**Figure 1:**
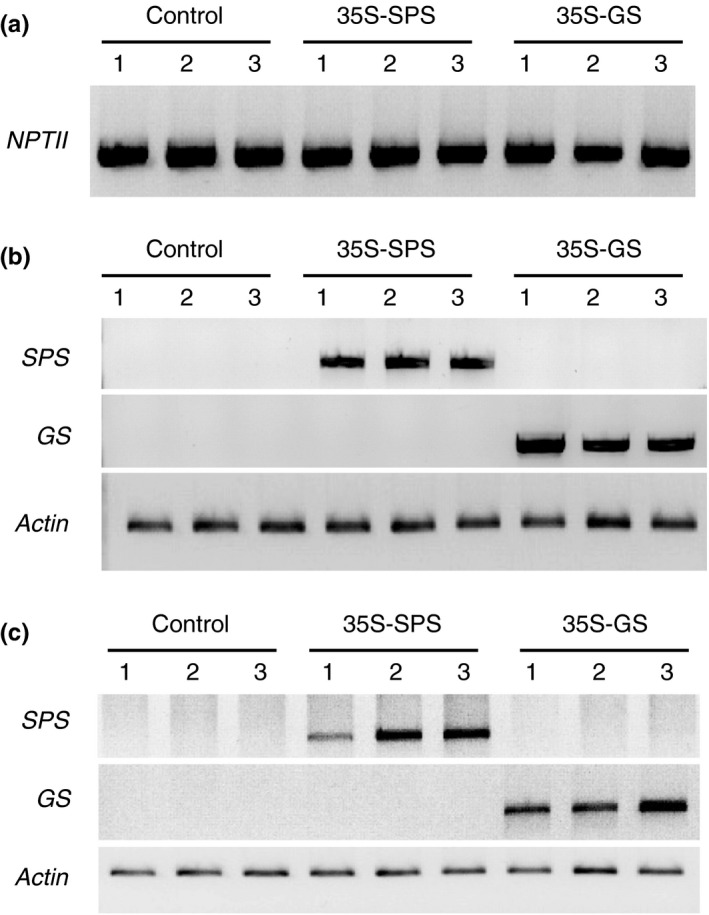
Analysis of the three classes of transformants: control (*Cambia 2300*), *35S‐SPS*, and *35S‐GS* plants for the integration and functionality of the gene constructs. (a) DNA isolated from three independent transformants for each class was isolated and subjected to genomic PCR using a *NPTII* specific primer set. The products were then fractionated on agarose gels. (b) RNA isolated from the leaves of the same plants used in panel A were subjected to RT‐PCR using primer sets specific for the *SPS* transgene, the *GS* transgene, and *Actin* and the products were electrophoresed. (c) RNA isolated from the nodules of the same set of plants used in panels A and B were subjected to RT‐PCR using primer sets specific for *SPS* transgene, *GS* transgene, and *Actin* and the products were subjected to electrophoresis

DNA isolated from three independent transformants representing the three sets of transformants: *CAMBIA2300* (control), the *CaMV 35S* promoter driving the maize *SPS* gene (*35S‐SPS*), and the *CaMV35S* promoter driving a soybean *GS*_*1*_ gene (*35S‐GS*) were subjected to PCR amplification using *NPTII* gene‐specific primers followed by electrophoresis. *NPTII* is the selectable marker in the tDNA conferring resistance to the antibiotic kanamycin. As seen in Figure 1, all the putative transformants selected for this study showed an amplicon of the expected size (800 bp), confirming that the putative transformants were indeed transformed. These plants were selected for further analysis. The three independent transformants representing the three classes were propagated vegetatively and the cuttings were inoculated with *S. meliloti*.

To check if the gene constructs are functional and that the promoter *CaMV35S* was functioning in a constitutive manner, we examined the plants for the expression of the transgenes in the leaves and nodules. RNA isolated from the leaves and nodules of the three classes of plants (control, *35S‐SPS*, and *35S‐GS*) was subjected to RT‐PCR using the two transgene specific primer sets along with the *Actin* primer set that was used as an internal control for RNA loads. The products along with known molecular weight DNA markers were electrophoresed on agarose gel. The *35S‐SPS* transformants showed an amplicon of the expected size (800 bp) and the *35S‐GS* transformants showed a 750‐bp amplicon in both the leaves and nodules. The control plants did not show an amplicon with either set of primers (Figure 1). These results confirm that the transgene was being transcribed in both the leaves and nodules.

### 3.2 The *35S‐GS* transformants along with the *35S‐SPS* transformants showed an increase in SPS level in their leaves

**Figure 2:**
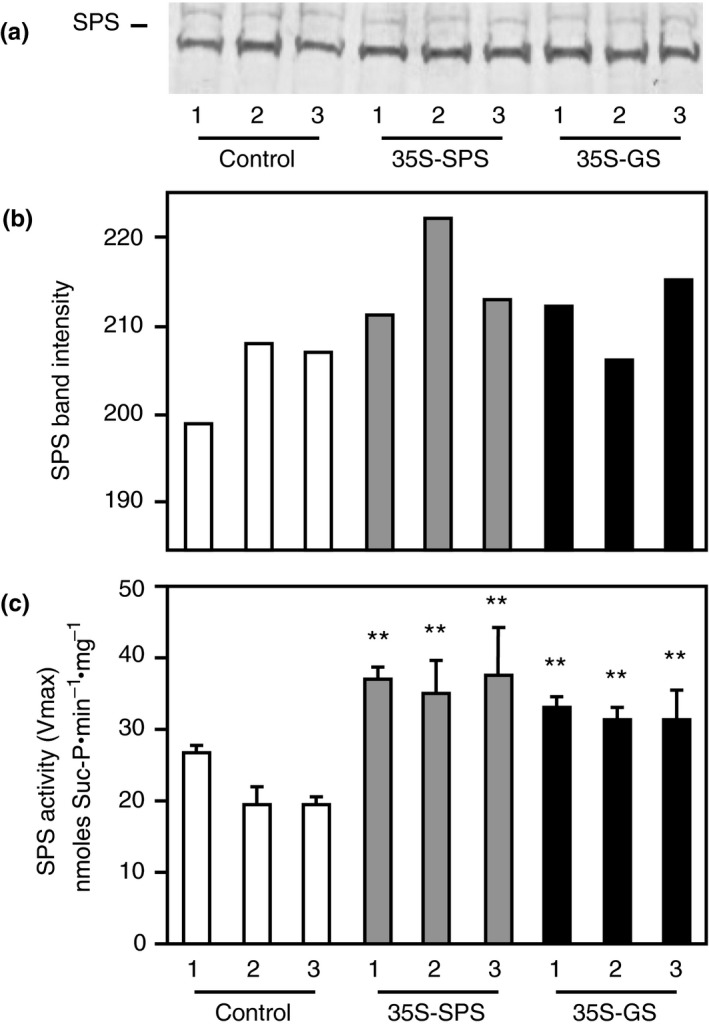
Analysis of SPS protein and SPS enzyme activity in the leaves of control, *35S‐SPS*, and *35S‐GS* transformants. (a) A quantity of 70 μg of total protein extracted from the leaves of three clonally propagated plants for each independent transformant was subjected to SDS–PAGE (7.5% acrylamide) followed by western blot analysis using SPS antibodies. A representative blot is shown here. The size of the immunoreactive band was determined based on the migration of proteins of known molecular weight. (b) The immunoreactive bands from the western blot were quantified using the KODAK image analysis software and plotted as band intensity. (c) The same leaf extracts used for western blot analysis were used for enzyme activity measurement by quantifying the synthesis of Suc‐6P from UDP‐Glc and Fru‐6P. SPS enzyme activity values are plotted as nmol Sucrose‐P mg^−1^ protein min^−1^. Values are the means ± *SD* of samples from three different replicates for each independent transformant. Significant differences from the average value obtained for the control plants were evaluated by ANOVA contrast test and shown by asterisks (*p <* *0.05 or **<0.01)

Western blot analysis was performed on total protein extracted from the leaves of three clonally propagated plants for each of the three independent transformants representing the three classes of plants using SPS antibodies. A representative blot with samples from one of the replicates for each individual transformant is shown in Figure 2a. The intensity of the immunoreactive bands from this blot was measured and plotted as bar graphs (Figure 2b). An immunoreactive band (~140 kD) was seen in all the samples, but the band intensity in the samples from both the *35S‐SPS* and *35S‐GS* transformants, in general, was higher when compared to controls. While the increase in SPS level in the *35S‐SPS* transformants over the control plants can probably be an attribute of the expression of the SPS transgene, the increase in SPS levels in the *35S‐GS* transformants over the control plants can only be ascribed to an increase in the endogenous SPS level.

Total soluble protein extracted from the leaves of the same plants as used for western blot analysis was subjected to the SPS enzyme activity assay. The enzyme activity was measured by quantifying the synthesis of Suc‐6P from UDP‐Glc and Fru‐6P, and SPS enzyme activity values were plotted as nmol Suc‐P mg^−1 ^protein min^−1^. The bars represent the average of the activities of the three replicates for each of the three independent transformants representing each of the three classes of plants (Figure 2c). SPS activity was significantly higher in the leaves of both the *35S‐SPS* and *35S‐GS* transformants when compared to the samples from the control plants with the levels being higher in the *35S‐SPS* transformants. These results indicate that the SPS activity in the leaves match the accumulation pattern of the corresponding protein.

### 3.3 The *35S‐GS* transformants showed accumulation of the GS_1_ transgene product in the leaves

**Figure 3:**
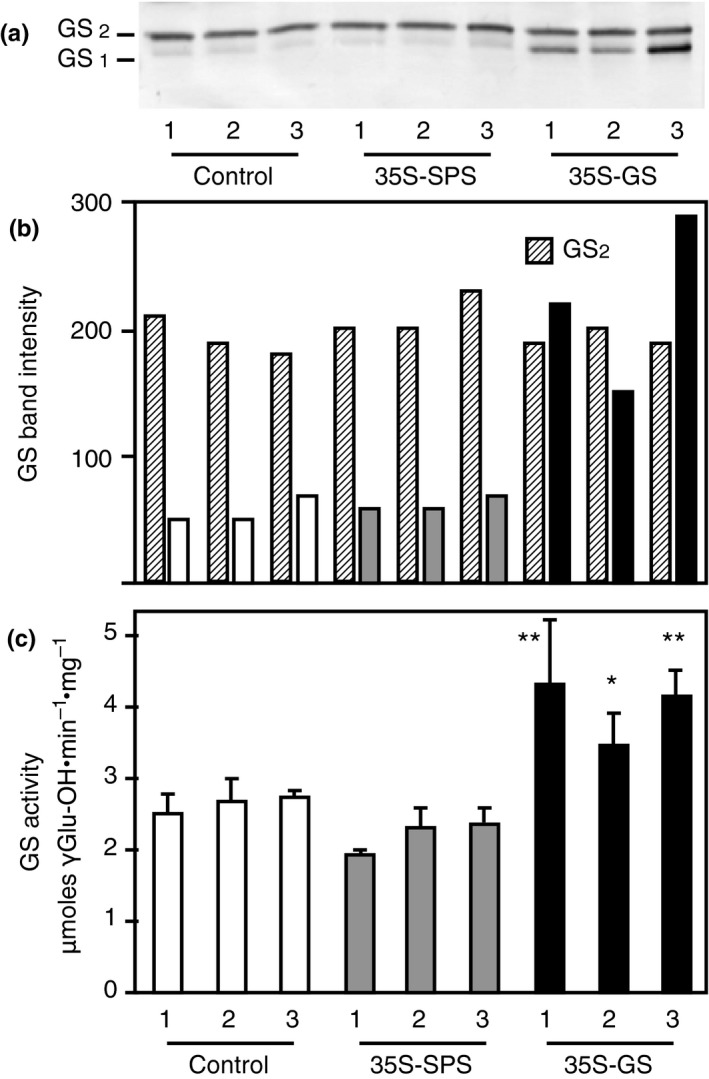
Analysis of GS protein and GS enzyme activity in the leaves of control, *35S‐SPS*, and *35S‐GS* transformants. (a) A quantity of 10 μg of total protein extracted from the leaves of three clonally propagated plants for each independent transformant was subjected to SDS–PAGE (10% acrylamide) followed by western blot analysis using GS antibodies. A representative blot is shown here. The size of the immunoreactive band was determined based on the migration of proteins of known molecular weight. (b) The immunoreactive bands from the western blot were quantified using the KODAK image analysis software and plotted as band intensity. (c) GS transferase activity was measured in the leaf extracts used for GS western blot analysis and GS activity values were plotted as μmol γ‐glutamyl hydroxamate produced per minute per mg of protein at 30°C. Values are the means ± *SD* of samples from three different replicates for each independent transformant. Significant differences from the average value obtained for the control plants were evaluated by ANOVA contrast test and shown by asterisks (*p <* *0.05 or **<0.01)

Western blot analysis using the GS antibodies was performed on total protein extracted from the leaves of the same three clonally propagated plants for each of the three independent transformants representing each of the three classes, as used for the analysis of SPS protein (Figure 2). A representative blot with samples from a replicate of each individual transformant is shown in Figure 3a. The intensity of the immunoreactive bands from this blot was measured and plotted as bar graphs (Figure 3b). An immunoreactive band corresponding to GS_2_ was seen in all the lanes and the intensity of the band was the same across all the lanes. A major immunoreactive band, migrating faster than the GS_2_ protein band was seen only in the lanes with leaf protein from the *35S‐GS* transformants, most likely representing the transgene product. All the lanes, besides showing the major GS_2_ band also showed an immunoreactive band migrating slightly slower than the band corresponding to the soybean GS_1_ (transgene) protein, though the band in the *35S‐GS* transformants appeared to be masked by the stronger transgene protein band. This band probably represents the endogenous GS_1_ protein.

The same samples used for western blotting were subjected to GS activity measurement using the transferase assay. The enzyme activity for the three replicates for each transformant was averaged and plotted as μmol γ‐glutamyl hydroxamate produced per minute per mg of protein. (Figure 3c). GS activity was significantly higher in the *35S‐GS* transformants compared to the activities in the *35S‐SPS* transformants and the control plants. One of the three *35S‐SPS* transformants had lower GS activities than the control plants. The overall means of control plants and *35S‐SPS* transformants were not different, and both means were highly statistically different from the *35S‐GS* transformants. These results indicate that the GS activity was higher in the *35S‐GS* transformants compared to the activity in the *35S‐SPS* transformants and control plants, which can be attributed to the accumulation of the transgene polypeptide.

### 3.4 The expression of *SPSB* gene was upregulated in the leaves of both the *35S‐SPS* and *35S‐GS* transformants

**Figure 4:**
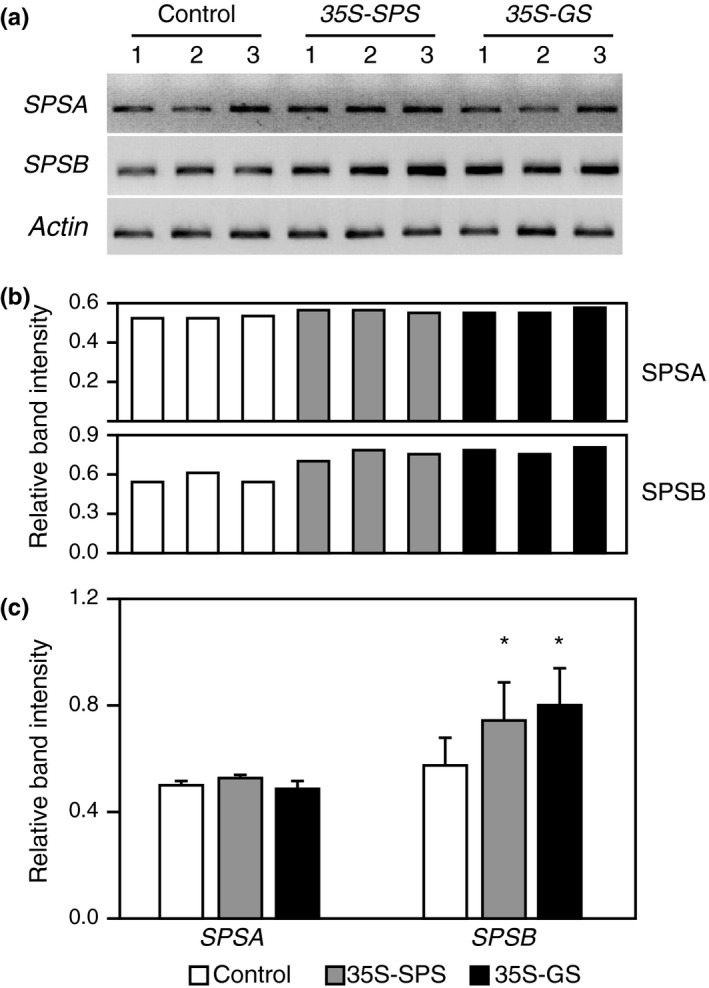
Analysis of transcript accumulation for the endogenous *SPS* genes in the leaves of control, *35S‐SPS*, and *35S‐GS* transformants. (a) A quantity of 2 μg of total RNA isolated from the leaves of the same set of plants used for protein analysis was subjected to semiquantitive RT‐PCR using primer sets specific for *SPSA*,*SPSB*, and *Actin* (internal control for RNA concentration). Following amplification, the products were subjected to electrophoresis. Data from a representative experiment is shown here. (b) The band intensities were measured using the KODAK image software. The ratio of the band intensity obtained for *SPSA* and *SPSB* genes relative to the band intensity obtained with the *Actin* primer set was calculated for each transformant. The values obtained for the three independent transformants representing the *35S‐SPS* and *35S‐GS* classes were then compared with the average value obtained for the three control plants and plotted as bar graphs. Significant differences from the control plants were evaluated by ANOVA contrast test and shown by asterisks (*p <* *0.05 or **<0.01)

To check if an increase in the accumulation of the SPS proteins in the leaves of the *35S‐GS* transformants is due to the induction of endogenous *SPS* genes, semiquantitative RT‐PCR was conducted on the RNA isolated from the leaves of the same plants that were used for western blot analysis (Figures 2 and 3) using primer sets for *SPSA* and *SPSB*. The number of cycles was fine tuned to ensure that the reaction was terminated during the log phase of amplification. *Actin* primers were also used to amplify *Actin* transcripts, used as an internal control for RNA concentration. The PCR products were subjected to electrophoresis and the band intensity for all the amplicons was measured and the values were adjusted based on the band intensity of the amplicon for *Actin*. All the three independent transformants for *35S‐SPS* and the *35S‐GS* classes, showed an increase in the amplicon intensity for *SPSB* over control. The value for each class of transformants (control, *35S‐SPS*, and *35S‐GS*) was then calculated as the average of the values for the three independent transformants (Figure 4). A significant increase in the level of the amplicon for *SPSB* was seen in the leaves of both the *35S‐SPS* and *35S‐GS* transformants when compared to the control plants. No difference in the amplicon levels for *SPSA* was seen among the three classes of transformants. This analysis was done on RNA from different replicates with the same overall outcome; both the *35S‐SPS* and *35S‐GS* transformants showed an increase in the *SPSB* transcript level when compared to the control plants.

### 3.5 The *35S‐SPS* and *35S‐GS* transformants showed higher Suc and starch content in their leaves compared to the control plants

With the rationale that an increase in SPS activity could translate into higher rates of Suc synthesis, Suc content was measured in the leaves of all the three classes of transformants. Total carbohydrates were extracted from the leaves of three clonally propagated plants for each transformed line and the average of these was then calculated (Table 2A). The average Suc content in the leaves of the *35S‐GS* transformants and *35S‐SPS* transformants were compared with the average Suc concentration for all the three control plants. All three individuals representing the two classes of transformants showed a statistically significant (50%–70%) increase in the Suc content in their leaf tissue compared to the leaves of control plants. These results indicate that the increase in SPS activity in the leaves of both the *35S‐SPS* and *35S‐GS* transformants is accompanied by an increase in Suc level.

**Table 2:** Analysis of forage quality of control, *35S‐SPS*, and *35S‐GS* transformants

(A)			
Genotype	Avg. lignin % dry basis	Avg. ADF % dry basis	Avg. NDF % dry basis
Control 1	4.82 ± 0.07	23.6 ± 5.07	29.21 ± 2.24
Control 2	5.24 ± 0.14	26.56 ± 0.3	31.95 ± 2.58
Control 3	4.82 ± 0.035	27.29 ± 0.05	31.44 ± 0.65
*35S‐SPS 1*	4.35 ± 0.03*	22.9 ± 0.2*	25.32 ± 0.93**
*35S‐SPS 2*	4.21 ± 0.11*	21.94 ± 0.411*	25.97 ± 0.13**
*35S‐SPS 3*	4.16 ± 0.12*	22.47 ± 0.96*	26.04 ± 0.16**
*35S‐GS 1*	4.50 ± 0.127*	23.91 ± 0.07	29.62 ± 0.02
*35S‐GS 2*	4.70 ± 0.06*	22.01 ± 1.97**	25.51 ± 1.41**
*35S‐GS 3*	4.22 ± 0.12*	22.67 ± 0.30*	24.50 ± 1.30**

(B)			
Genotype	Avg. CP % dry basis	Avg. RFQ	Avg. RFV
Control 1	20.16 ± 0.64	204.66 ± 4.5	197.33 ± 2.51
Control 2	20.77 ± 0.20	210 ± 11.7	200.33 ± 15.5
Control 3	19.89 ± 0.78	229.5 ± 4.5	198 ± 6
*35S‐SPS 1*	24.24 ± 0.7**	253.66 ± 4.7**	253.33 ± 1.52**
*35S‐SPS 2*	23.84 ± 0.17**	248 ± 2.0**	251.33 ± 0.57**
*35S‐SPS 3*	24.84 ± 0.23**	259 ± 2.0**	258.66 ± 1.1.52**
*35S‐GS 1*	22.01 ± 0.70**	213.33 ± 4.16	225.5 ± 4.5**
*35S‐GS 2*	22.47 ± 0.06**	238.33 ± 3.51**	245.16 ± 1.7**
*35S‐GS 3*	22.08 ± 0.69**	252 ± 3.0**	257 ± 2.0**

The clonally propagated plants was grown 50 days post‐inoculation at which time they were all cut down to the base and grown till the onset of flowering. The shoots were then cut at the base and sent out to the SDK labs for forage quality analysis. The control plants were cut ~2 weeks later since they flowered late compared to the other two classes of transformants. (A) The lignin content, acid detergent fiber (ADF), and neutral detergent fiber (NDF) were measured. Values for three different replicates for each independent transformant were measured, and the mean value ± *SD* was calculated for each plant. Significant differences between the *35S‐SPS* and *35S‐GS* from the average value obtained from the control plants were evaluated by ANOVA contrast test and shown by asterisks (^*^ *p *<* *0.05 or ^**^<0.01). (B) Relative forage quality (RFQ), relative feed value (RFV), and crude protein (CP) content were measured. The RFQ and RFV were calculated based on fiber and protein content. Values for three different replicates for each independent transformant representing each class were measured, and the mean value ± *SD* was calculated for each plant. Significant differences between the *35S‐SPS* and *35S‐GS* from the average values obtained for the control plants were evaluated by ANOVA contrast test and shown by asterisks (^*^ *p *<* *0.05 or ^**^<0.01).

Starch was isolated from the same samples as used for Suc extraction as described in Materials and Methods. To measure starch content, it was digested with α‐amyloglucosidase to release the Glc units and the Glc content was measured spectrophotometrically. The starch content like the Suc content was measured in the leaves of the three clonally propagated plants for each transformed line and the average of these values was then calculated. The average starch content in each independent transformant for the two classes, *35S‐SPS* and *35S‐GS*, was then compared with the average value for the three independent control plants. The starch content in both classes, *35S‐SPS* and *35S‐GS* transformants, showed significantly higher level compared to the control plants with the level being higher in the *35S‐SPS* transformants (Figure 8b). The results suggest that there is higher conversion of Suc to starch in the leaves of the *35S‐SPS* transformants compared to the leaves in the *35S‐GS* transformants.

### 3.6 Both the *35S‐SPS and 35S‐GS* transformants showed higher accumulation of SPS protein and enzyme activity in their nodules compared to the control plants

**Figure 5:**
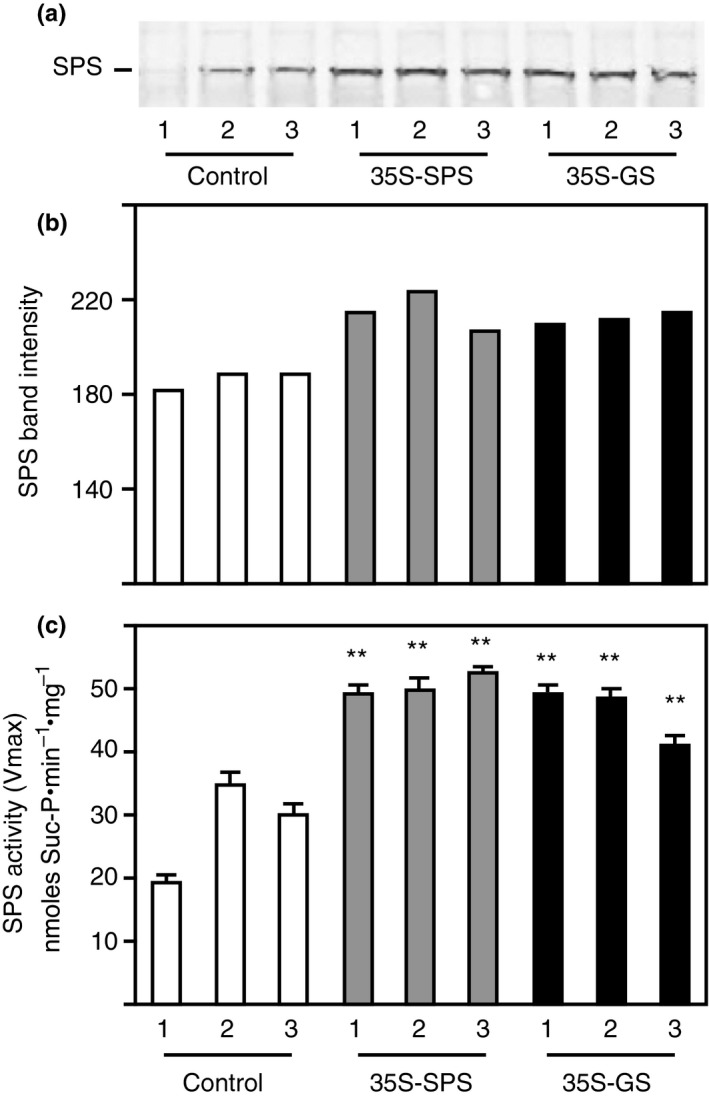
Analysis of SPS protein and SPS enzyme activity in the nodules of control, *35S‐SPS* and *35S‐GS* transformants. (a) A quantity of 50 μg of total protein extracted from the nodules of three clonally propagated plants for each independent transformant was subjected to SDS–PAGE (7.5% acrylamide) followed by western blot analysis using SPS antibodies. A representative blot is shown here. The size of the immunoreactive band was determined based on the migration of proteins of known molecular weight. (b) The immunoreactive bands from the western blot were quantified using the KODAK image analysis software and plotted as band intensity. (c) The same nodule extracts used for western blot analysis were used for enzyme activity measurement by quantifying the synthesis of Suc‐6P from UDP‐Glc and Fru‐6P. SPS enzyme activity values are plotted as nmol Suc‐P mg^−1 ^protein min^−1^. Values are the means ± *SD* of samples from three different replicates for each independent transformant. Significant differences from the average value obtained for the control plants were evaluated by ANOVA contrast test and shown by asterisks (*p <* *0.05 or **<0.01)

To check how constitutive expression of *SPS* and *GS*_*1*_ transgenes affects the accumulation of the SPS proteins in the nodules, total protein extracted from the nodules of three clonally propagated plants for each of the three independent transformants for each of the three classes of plants was subjected to western blot analysis using the SPS antibodies. A representative blot with samples from one replicate for each transformant is shown in Figure 5a. The intensity of the immunoreactive bands was measured in each case and plotted as bar graphs (Figure 5b). An immunoreactive band (~140 kD) was seen in all the samples, but the band intensity in all the samples from both the *35S‐SPS* and *35S‐GS* transformants, was higher when compared to controls (Figure 5a,b). While the increase in SPS level in the *35S‐SPS* transformants over the control plants can probably be an attribute to the expression of the SPS transgene, the increase in SPS levels in the *35S‐GS* transformants over the control plants can only be ascribed to an increase in the endogenous SPS level.

The samples used for western blot analysis were used for measuring SPS enzyme activity by quantifying the synthesis of Suc‐6P from UDP‐Glc and Fru‐6P and SPS enzyme activity values was plotted as nmol Suc‐P mg^−1 ^protein min^−1^. The bars represent the average of the activities of the three replicates for each of the three independent transformants for each of the three classes of plants (Figure 5c). SPS activity was significantly higher in the nodules of both the *35S‐SPS* and *35S‐GS* transformants when compared to the samples from control plants. The results on enzyme activity closely follows SPS protein accumulation pattern, suggesting that the increase in SPS levels and SPS enzyme activity in the nodules of the *35S‐GS* transformants is probably due to the activation of endogenous *SPS* gene/s.

### 3.7 Both the *35S‐SPS and 35S‐GS* transformants showed higher accumulation of GS protein and GS enzyme activity in their nodules compared to the control plants

**Figure 6:**
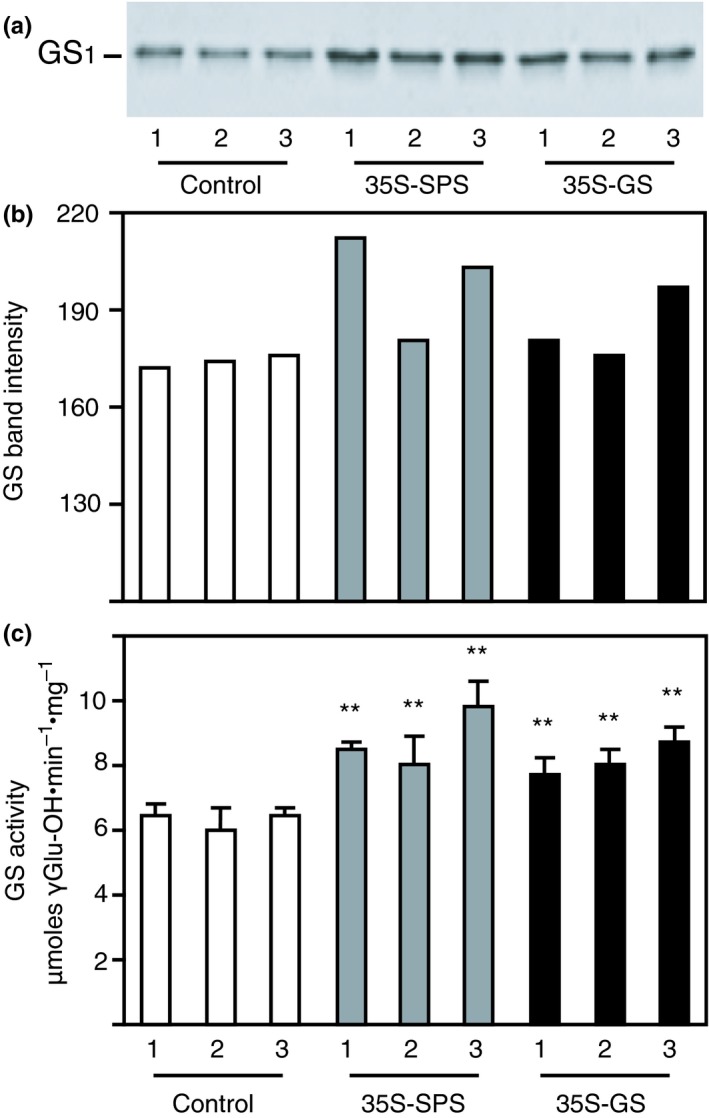
Analysis of GS protein and GS enzyme activity in the nodules of control, *35S‐SPS*, and *35S‐GS* transformants. (a) A quantity of 1 μg of total protein extracted from the leaves of three clonally propagated plants for each independent transformant was subjected to SDS–PAGE (10% acrylamide) followed by western blot analysis using GS antibodies. A representative blot is shown here. The size of the immunoreactive band was determined based on the migration of proteins of known molecular weight. (b) The immunoreactive bands from the western blot were quantified using the KODAK image analysis software and plotted as band intensity. (c) GS transferase activity was measured in the nodule extracts used for GS western blot analysis and GS activity values were plotted as μmol γ‐glutamyl hydroxamate produced per minute per mg of protein at 30°C. Values are the means ± *SD* of samples from three different replicates for each independent transformant. Significant differences from the average value obtained for the control plants were evaluated by ANOVA contrast test and shown by asterisks (*p <* *0.05 or **<0.01)

Western blot analysis using the GS antibodies was performed on total protein extracted from the nodules of the same three clonally propagated plants for each of the three independent transformants, as used for analysis of SPS protein (Figure 5). A representative blot with samples from one replicate for each transformant is shown in Figure 6a. The intensity of the immunoreactive bands was measured in each case and plotted as bar graphs (Figure 6b). As expected, all the samples showed an immunoreactive band of 39 kD, with the GS antibodies; however, both the *35S‐SPS* and *35S‐GS* transformants showed higher accumulation of the immunoreactive band when compared to the control plants with the levels being higher in the *35S‐SPS* transformants (Figure 6a,b).

The same samples used for western blotting were subjected to GS enzyme activity measurement using the transferase assay. The enzyme activity for the three replicates for each individual transformant was averaged and plotted as μmol γ‐glutamyl hydroxamate produced per minute per mg of protein. (Figure 6c). GS activity was significantly higher in both the *35S‐SPS and 35S‐GS* transformants compared to the activities in the control plants. Taken together, it appears that the GS enzyme activity level is consistent with GS protein accumulation in the nodules. What is most intriguing is that both GS protein accumulation and GS enzyme activity was higher in the nodules of the *35S‐SPS* transformants compared to the *35S‐GS* transformants, implying that there is an induction of *GS*_*1*_ gene(s) in the nodules of the *35S‐SPS* transformants.

### 3.8 The expression of the endogenous *SPS* and *GS*_*1*_ gene members were upregulated in the nodules of both the *35S‐SPS* and *35S‐GS* transformants

**Figure 7:**
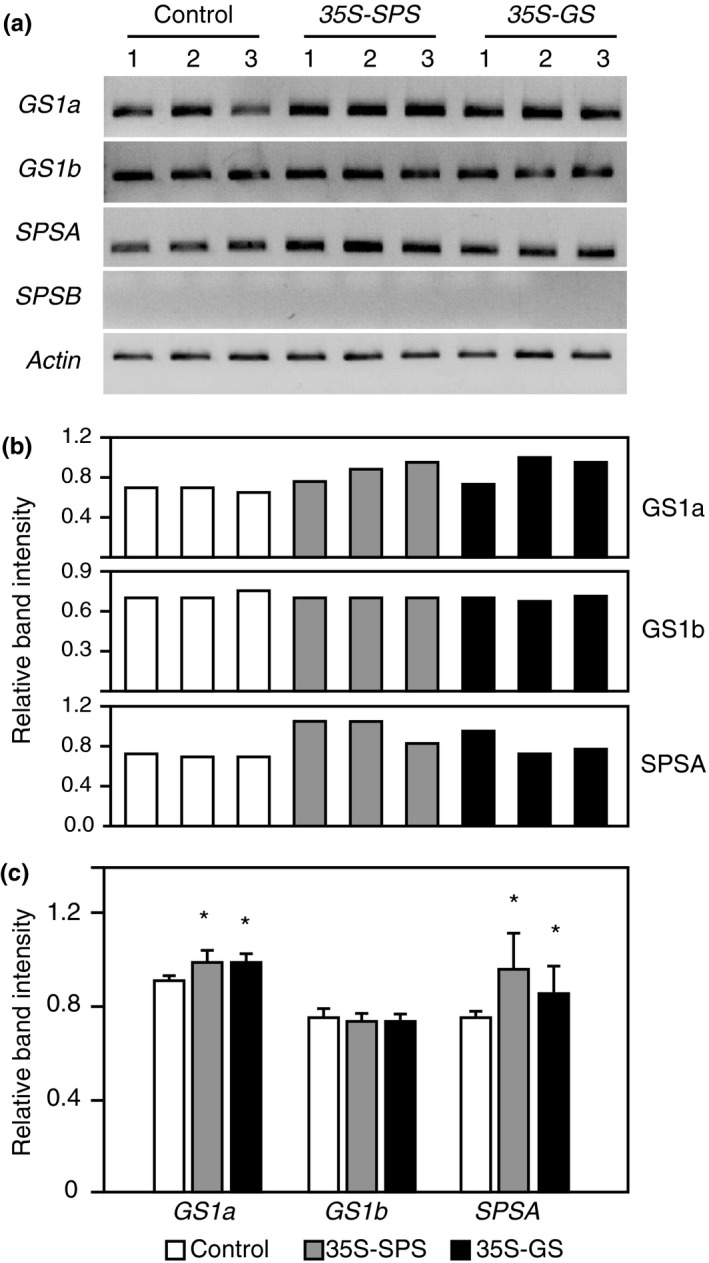
Analysis of transcript accumulation for the endogenous *SPS* genes in the nodules of Control, *35S‐SPS*, and *35S‐GS* transformants. (a) A quantity of 2 μg of total RNA isolated from the nodules of the same set of plants used for protein analysis was subjected to semiquantitive RT‐PCR using primer sets specific for *GS1a*,*GS1b*,*SPSA*,*SPSB*, and *Actin* (internal control for RNA concentration). Following amplification, the products were subjected to electrophoresis. Data from a representative experiment are shown here. (b) The band intensities were measured using the KODAK image software. (c) The ratio of the band intensity obtained for *GS1a*,*GS1b*,*SPSA*,*SPSB* genes relative to the band intensity obtained with the *Actin* primer set was calculated for each transformant. The values obtained for the three independent transformants representing the *35S‐SPS* and 35S‐GS classes were then compared with the average value obtained for the three control plants and plotted as bar graphs. Significant differences from the control plants were evaluated by ANOVA contrast test and shown by asterisks (*p <* *0.05 or **<0.01)

An increase in the accumulation of SPS proteins in the nodules of the *35S‐GS* transformants and that of GS in the nodules of the *35S‐SPS* transformants prompted us to check the expression level of the endogenous *SPS* and *GS*_*1*_ genes. Semiquantitative RT‐PCR was performed on the RNA isolated from the nodules of the same plants used for western blot analysis using primer sets for *GS1a, GS1b, SPSA*, and *SPSB*. The number of cycles was fine tuned to ensure that the reaction was terminated during the log phase of amplification. *Actin* primers were also used to amplify the *Actin* transcript as an internal control for RNA concentration. The products, following PCR, were subjected to electrophoresis and the band intensity for all the amplicons was measured and the values were adjusted based on the band intensity of the amplicon for *Actin*. This analysis was done on RNA isolated from multiple replicates and the results of a representative experiment are shown. The bar graph shows the intensity of the bands for the amplification products (Figure 7b). No amplicon was obtained with the *SPSB* primer set consistent with the fact that *SPSB* is not expressed in the nodules (Aleman et al., 2010). All the three independent transformants for both the *35S‐SPS* and *35S‐GS* classes showed an increase in the amplicon level for *SPSA* and *GS1a* in their nodules when compared to the control. The value for each class of transformants (control, *35S‐SPS*, and *35S‐GS*) was then calculated as the average of the values for the three independent transformants. A significant increase in the amplicon level for *SPSA* and *GS1a* was seen in both the *35S‐SPS* and *35S‐GS* transformants when compared to the control plants with the levels in the *35S‐SPS* transformants superseding the levels in the *35S‐GS* transformants (Figure 7). No difference in the amplicon level for *GS1b* was seen across all the samples. The results indicate that the expression of the gene members *SPSA* and *GS1a* are upregulated in the nodules of both the *35S‐SPS* and *35S‐GS* transformants, more so in the nodules of the *35S‐SPS* transformants.

### 3.9 The *35S‐SPS* and *35S‐GS* transformants showed higher Suc and starch content in their nodules compared to the control plants

**Figure 8:**
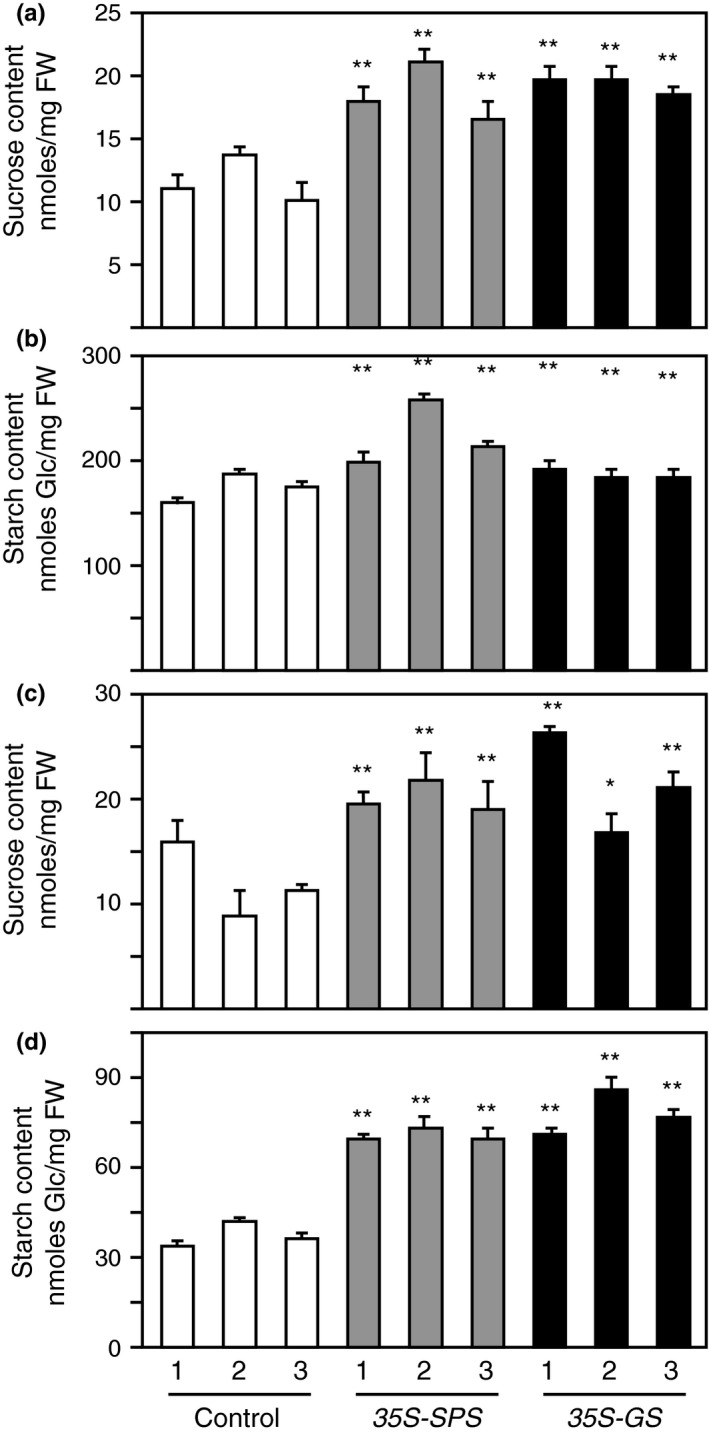
Sucrose and starch content in the leaves and nodules of control, *35S‐SPS*, and *35S‐GS* transformants. Sucrose and starch content were measured in the leaves (a, b) and nodules (c, d) as described in Section 2. Sucrose content was plotted as nmoles Suc mg^−1^ fresh weight (fwt) and starch content was plotted as nmoles Gluc mg^−1^ fresh weight (fwt). Values for three replicates for each of the three independent transformants representing each class were measured, and the mean value ± *SD* was calculated for each transformant. Significant differences between the *35S‐SPS* and *35S‐GS* transformants from the average value obtained from the three control plants were evaluated by ANOVA contrast test and shown by asterisks (*p <* *0.05 or **<0.01)

The Suc content was measured in the nodules, the same way as for leaves. The Suc content in the nodules of three replicates for each independent transformant was measured and the average value for each of the three independent transformants for each of the three classes was tabulated as average Suc content (Figure 8c).

Starch was isolated from the same samples as used for Suc extraction. The starch content like the Suc content was measured in the nodules of the three clonally propagated plants for each independent transformant representing the three classes of plants. The starch content in the nodules of three replicates for each independent transformant was measured and the average value for each of the three independent transformants representing each of the three classes was plotted as average starch content (Figure 8d).

The average values for Suc and starch for each of the three independent transformants representing each of the *35S‐SPS* and *35S‐GS* classes were compared with the average Suc and starch content in the three independent control plants. Both the Suc and starch concentration in all the three independent transformants representing the *35S‐SPS* and *35S‐GS* classes showed a significant increase of ~60%–70% in Suc content and up to 100% in starch content (Figure 8c,d). The results would indicate that there is more Suc transported into the nodules of the transformants and a high proportion is converted into starch. The higher levels of SPS activity in the nodules may play a role in the synthesis of starch by increasing Suc cycling.

### 3.10 The *35S‐SPS* and *35S‐GS* transformants showed higher level of the key enzymes involved in ammonia assimilation in their nodules compared to the control plants

Sucrose imported from the leaves to the nodules is acted upon by SucS to produce hexoses and hexose phosphates which are metabolized through the glycolytic pathway to produce phosphoenolpyruvate (PEP). PEP carboxylase (PEPC) and malate dehydrogenase (MDH) activities divert C flux from glycolysis to form malate which is the primary source of C transported into the symbiosomes that houses the bacteroids (White et al., 2007). The pyruvate derived from PEP enters the Krebs cycle to produce α‐KG. GOGAT in the GS‐GOGAT cycle combines Gln and α‐KG to produce Glu which then provides the C skeletons for loading up with ammonia, the reaction being catalyzed by GS. Glutamine can then be converted into asparagine (Asn); one of the enzymes in this conversion is asparagine synthetase (AS).

**Figure 9:**
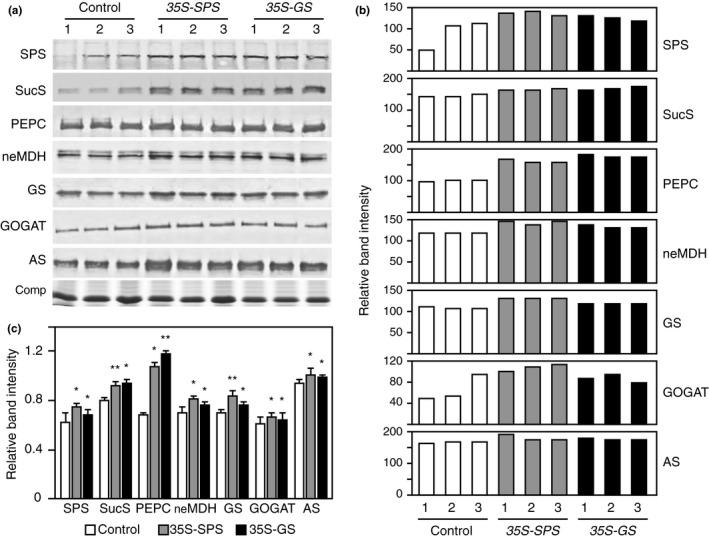
Examination of the steady‐state levels of key enzymes in C and N metabolic pathways in the nodules of control, *35S‐SPS*, and *35S‐GS* transformants. (a) Protein extracts from the nodules were subjected to SDS–PAGE followed by western blot analysis using the different antibodies (as indicated). A Coomassie blue stained (Comp) region of the protein gel is shown here as a reference for protein loads. The MW in kD indicated for each panel were based on the migration of known molecular weight markers. (b) The immunoreactive bands were quantified using the KODAK image analysis software, and the band intensity was normalized to the Coomassie stained endogenous protein band (Comp). The normalized band intensity was the plotted for each individual transformant representing the three classes. (c) The average of the values obtained for the three independent transformants representing the *35S‐SPS* and 35S‐GS classes were then compared with the average value obtained for the three control plants and plotted as bar graphs. The values are the means ± *SE* of samples from three different independent transformants (Panel A) (*n* = 3) for each class of transformants. Significant differences from the control plants were evaluated by ANOVA contrast test and shown by asterisks (*p <* *0.05 or **<0.01)

An increase in the amount of Suc imported in the nodules of the *35S‐SPS* and *35S‐GS* transformants would imply that there is an increase in the availability of substrates that is needed to support an increase in both N_2_ fixation and ammonia assimilation. One would then conjecture that an increase in these metabolic pathways would require increased enzyme level and or/enzyme activity for the different reactions in the pathways. To check for the levels of the different enzymes, western blot analysis was performed on nodule proteins from the three classes of plants. Protein extracted from the nodules of three independent transformants from each class was subjected to immunoblot analysis using antibodies for the different enzymes as shown in Figure 9a. As an internal control for protein loads, a panel from a gel with fractionated proteins stained with Coomassie blue is shown. The amount of protein loaded on the gels for each enzyme was adjusted to ensure that the band intensities were not saturated. Immunoreactive bands were seen with all the antibodies used in all the lanes (Figure 9a). The intensity of the immunoreactive bands was quantified and plotted. As seen in Figure 9b, in all cases, each of the three *35S‐SPS* and *35S‐GS* transformants showed higher level of band intensities when compared to the control plants. The average band intensity for each class of transformants was then calculated and subjected to statistical analysis. The values obtained for the *35S‐SPS* and *35S‐GS* transformants were compared with the average value obtained for the control plants.

Sucrose synthase, neMDH, and AS appear to be in the form of a doublet indicating that in each case there are two gene members that are expressed in the nodules. In each case, however, only one of the bands in the doublet showed an increase in the *35S‐SPS* and *35S‐GS* transformants. Both the *35S‐SPS* and *35S‐GS* transformants showed a significant increase in the levels of SPS, SucS, PEPC, neMDH, NADH‐GOGAT, GS_1_ and AS, when compared to the control plants. An increase in the SucS level would imply increased sink strength or enhanced Suc import from the leaves while an increase in the levels of the other enzymes would mean a boost in N_2_ fixation and the assimilation of ammonia.

### 3.11 Compared to the control plants, the *35S‐SPS* and *35S‐GS* transformants showed increased photosynthetic rates

**Figure 10:**
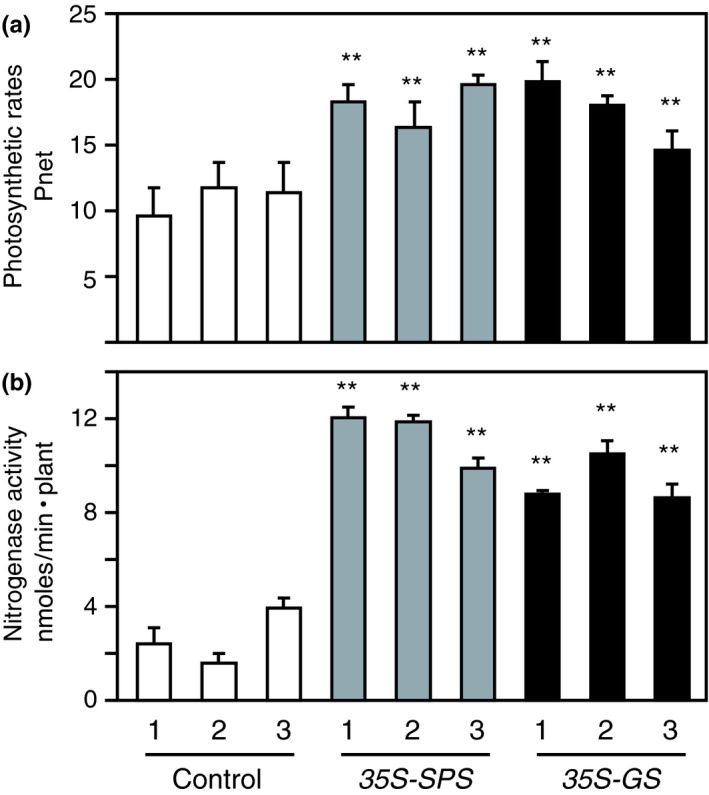
Analysis of (a) photosynthetic rates in the leaves and (b) nitrogenase activity in the nodules of control, *35S‐SPS* and *35S‐GS* transformants. (a) Clonally propagated plants were inoculated with *Sinorhizobium meliloti* and 30 days post‐inoculation, the photosynthetic rates were measured in mature trifoliate leaves from three replicates for each independent transformant. Photosynthetic rates (*P*_net_), measured as mmole CO_2_ m^−2^ s^−1^, were determined using a conifer chamber attached to a Li‐Cor 6400 photosynthesis system. Values for three different replicates for each independent transformants were calculated as mean value ± *SD*. Significant differences between each independent transformant belonging to the *35S‐SPS* and *35S‐GS* classes, and the average value obtained for the three control plants were evaluated by ANOVA contrast test and shown by asterisks (*p <* *0.05 or **<0.01). (b) Established cuttings were inoculated with *S. meliloti* and allowed to grow for a period of 30 days. The plants were then uprooted, and the roots of the plants were placed individually in mason jars and nitrogenase activity was measured as nmol ethylene per plant using the acetylene reduction assay as described in Section 2. Values for three to five different replicates for each independent transformants representing each class were measured, and the mean value ± *SD* was calculated for each individual transformant. Significant differences between the *35S‐SPS* and *35S‐GS* transformants from the average value obtained for the control plants were evaluated by ANOVA contrast test and shown by asterisks (*p <* *0.05 or **<0.01)

Tobacco plants overexpressing cytosolic *GS* have been shown to have higher photosynthetic rates (Fuentes et al., 2001; Seger et al., 2015), and similarly, overexpression of SPS has also been shown to be accompanied by an increase in photosynthetic rates (Baxter et al., 2003; Seger et al., 2015). To check if alfalfa plants overexpressing *SPS* and *GS*_*1*_ transgene show the same trend with regard to photosynthesis, we measured net photosynthetic rates (*P*_net_) under ambient (400 mmol mol^−1^) CO_2_ concentration in the control, *35S‐SPS*, and *35S‐GS* transformants. Three clonally propagated plants representing each independent transformant were used in these measurements, and the average of the values obtained from the three replicates was plotted (Figure 10a). The *P*_net_ values for each of the three transformants for the *35S‐SPS* and *35S‐GS* classes were compared with the average of *P*_net_ values for all the three control plants. The *P*_net_ rates were significantly higher in all the transformants belonging to both the *35S‐SPS* and *35S‐GS* classes.

### 3.12 Compared to the control plants, the *35S‐SPS* and *35S‐GS* transformants showed elevated nitrogenase activity

An increase in the MDH level in the nodules of the *35S‐SPS* and *35S‐GS* transformants could be an indicator of increased nitrogenase activity since malate is the form of organic acid used by the bacteroids (White et al., 2007). The root system of three replicates for each independent transformant representing all three classes of plants was assayed for nitrogenase activity using the ARA method as described in Section 2. The activity was measured as the amount of ethylene produced per plant and the values were plotted as nmoles min^−1^ plant^−1^. The average values for nitrogenase activity for each of the three independent transformants representing each of the three classes of plants were based on the values obtained from three to five replicates. Nitrogenase activity for the individual transformants belonging to the two classes, *35S‐SPS* and *35S‐GS*, was compared to the average values obtained for all the control plants. As seen in Figure 10b, all the transformants from each class showed significantly higher nitrogenase activity, with the level being higher in the *35S‐SPS* transformants.

### 3.13 Compared to the control plants, the *35S‐SPS* and *35S‐GS* transformants showed increased growth, biomass, and nodule number

**Figure 11:**
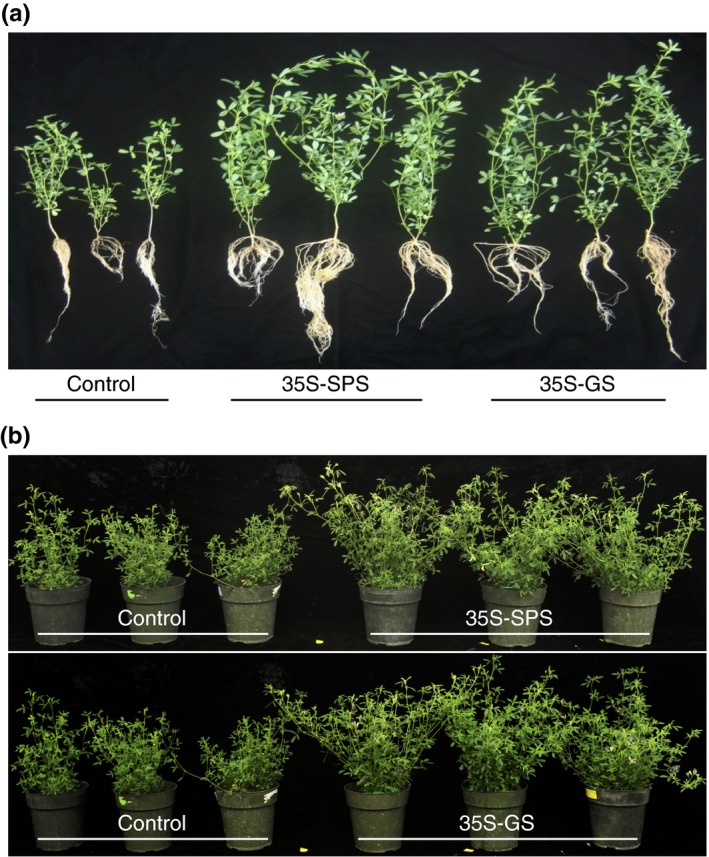
Visualization of growth in the control, *35S‐SPS*, and *35S‐GS* transformants at different stages. (a) Established transformants were used to obtain shoots for propagation. The cut shoots were planted on vermiculite and once established (~10 days) after the start day, the cuttings were inoculated with *Sinorhizobium meliloti*, and allowed to grow for a period of 30 days. The plants were uprooted and visualized. A plant representing each of the three independent transformant for each class were photographed. (b) Clonal replicates (2 per pot) were used for each individual transformant for each of the three classes of plants. The plants were grown till the onset of flowering and then cut down to the base. This process was repeated, and then, the plants were grown till the onset of flowering in the *35S‐SPS* and *35S‐GS* transformants, at which time they were photographed. The plants were arranged as: control and *35S‐SPS* (top panel), and control and *35S‐GS* transformants (bottom panel)

**Figure 12:**
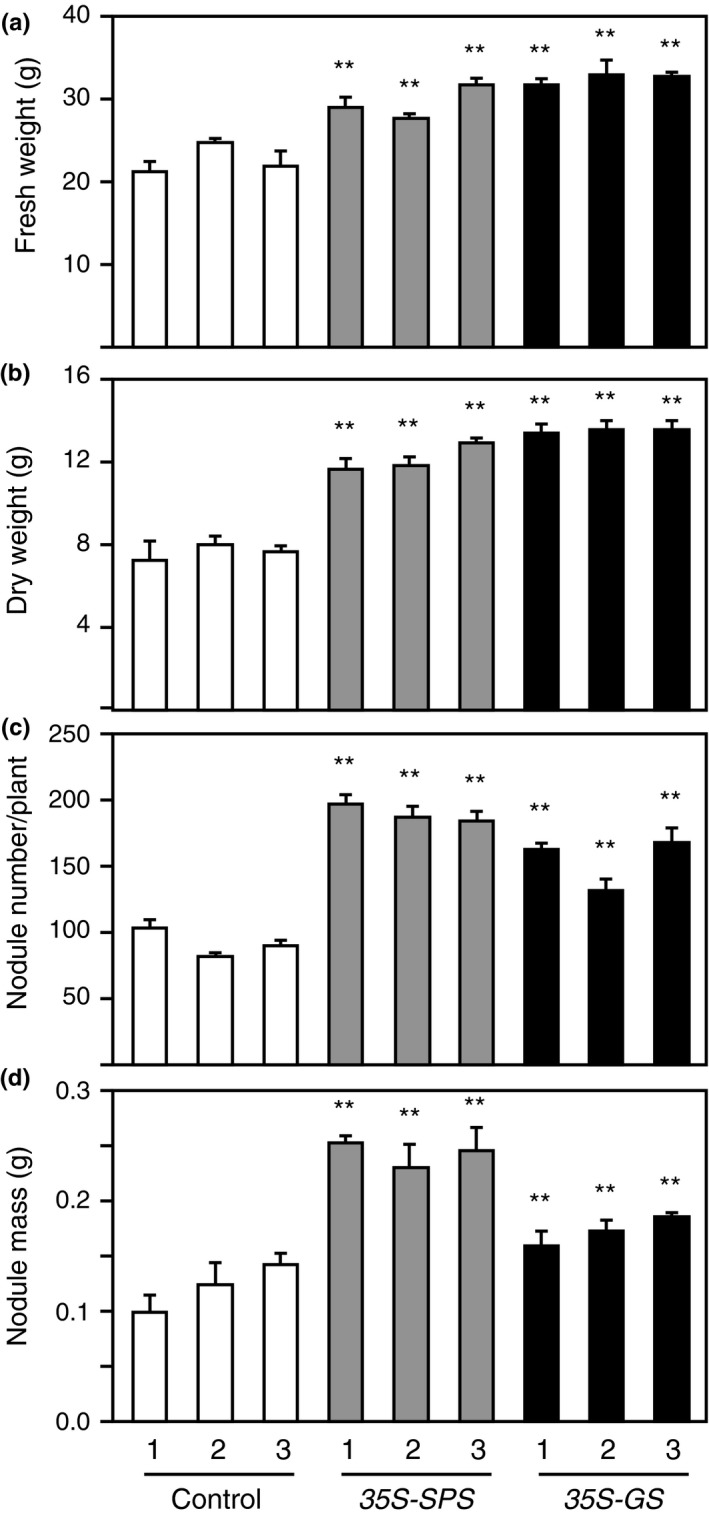
Analysis of fresh weight and dry weight of shoots and nodule number and mass of control, *35S‐SPS*, and *35S‐GS* transformants. (a, b) The plants from Figure 11, after being photographed, were cut down and allowed to grow back to just before the onset of flowering and were then cut at the base and weighed for fresh weight. For dry weight, the tissue was kept in paper bags for 2 weeks and then weighed. The control plants were cut ~2 weeks later since they flowered late compared to the other two classes of transformants. Values in grams for three different replicates for each independent transformants representing each class were measured, and the mean value ± *SD* was calculated for each individual transformant. Significant differences between the *35S‐SPS* and *35S‐GS* transformants from the average value obtained for the control plants were evaluated by ANOVA contrast test and shown by asterisks (*p <* *0.05 or **<0.01). (c, d) Established cuttings were inoculated with *Sinorhizobium meliloti* and allowed to grow for a period of 30 days. The plants were uprooted and the nodules were harvested. The nodules per plant were counted and weighed. Values in numbers/grams for three different replicates for each independent transformant representing each class were measured, and the mean value ± *SD* was calculated for each individual transformant. Significant differences between the *35S‐SPS* and *35S‐GS* transformants from the average value obtained for the control plants were evaluated by ANOVA contrast test and shown by asterisks (*p <* *0.05 or **<0.01)

Cuttings of the same size from the primary transformants were made and planted in pots (vermiculite), and were then inoculated with *S. meliloti*. Thirty days following inoculation, a set of cuttings were uprooted, and the vermiculite was washed off from the roots, and the whole plant with the root system was photographed (Figure 11a). The *35S‐SPS* transformants grew the fastest and had the most robust root system compared to the *35S‐GS* transformants, which in turn performed better than the control plants. The nodules were harvested, counted, and weighed. The number and weight of the nodules for each plant (three replicates) were analyzed and the average value for the three independent transformants representing each class of transformants was plotted as average nodule number and nodule mass per plant (Figure 12). The nodule number and weight followed the same trend as the shoot and root growth pattern. The *35S‐SPS* transformants showed the highest number of nodules and the nodules had a higher mass.

Another set of cuttings were grown for a longer period of time to check for the timing of flowering and measurement of fresh and dry weight of the shoots. The *35S‐SPS* transformants started flowering around 30 days post‐inoculation, the *35S‐GS* transformants started flowering about a week after the *35S‐SPS* plants while flowering in the control plants happened around 50 days post‐inoculation, about 2 weeks later than the *35S‐SPS* transformants. The plants were cut down to the base after 50 days of growth following inoculation, and allowed to regrow for 45 days and then photographed (Figure 11b). Both the *35S‐SPS* and *35S‐GS* plants outgrew the control plants and the flowering in the control plants was delayed by about 2 weeks. At this stage, both the *35S‐SPS* and *35S‐GS* transformants showed no difference in growth. After being photographed, the plants were cut down at the time of onset of flowering, as such the control plants were allowed to grow for another 15 days before being cut. The cut material was then weighed and put in bags for drying. The fresh and dry biomass were measured when the plants were mature (at the onset of flowering), and as seen in Figure 12c,d, both the fresh weight and dry weight of the *35S‐SPS* and *35S‐GS* plants were significantly higher than the control plants.

### 3.14 The *35S‐SPS* and *35S‐GS* transformants exhibited improved forage quality when compared to the control plants

Forage quality is defined as a measure of the potential of forage to produce a desired animal response. Forage quality is positively associated with protein content and negatively with fiber content. Laboratory analyses can be used to determine the nutritive value. In general, forage quality is calculated based on the protein and fiber content. The fiber content is classified into two categories: acid detergent fiber (ADF) and neutral detergent fiber (NDF). ADF includes cellulose and lignin while NDF is made up of the structural components including the cell walls. The lignin content was also measured by itself. The material from the same batch as used for biomass measurement was sent away to the SDK lab for analysis of forage quality (Table 2). The average values for lignin content, ADF, NDF, CP, RFQ, and RFV were calculated from the replicates (clonally propagated plants) for each individually transformed lines. The values for these parameters for the individual transformant belonging to the *35S‐SPS* and *35S‐GS* classes were compared to the average of the values obtained for all the three control plants.

Both the *35S‐SPS* plants and *35S‐GS* plants showed lower NDF and ADF content when compared to the control plants. While the *35S‐SPS* transformants showed a 19% drop in ADF and 14% in NDF, the *35S‐GS* transformants showed a drop of 15% in ADF and 10% in NDF, when compared to control. The insoluble lignin content in the *35S‐SPS* and *35S‐GS* transformants showed a 13% and 6% drop, respectively, when compared to the control plants. The crude protein content showed a ~20% increase in the *35S‐SPS* transformants and ~10% increase in the *35S‐GS* transformants. The relative forage quality (RFQ) in the *35S‐SPS* plants was 21% higher than the control plants, whereas the *35S‐GS* plants showed a 12% increase in forage quality. Moreover, the relative feed value (RFV) in the *35S‐SPS* and *35S‐GS* transformants was 20% and 10% higher compared to the control plants, respectively.

## 4 DISCUSSION

In this study, we have shown that the constitutive overexpression of *GS*_*1*_ improves plant growth and performance in alfalfa just as has been reported for alfalfa plants overexpressing *SPS* in a constitutive manner (Gebril et al., 2015). The question that we have raised in this study is why an increase in the expression of these two genes with completely different functions but with key roles in primary metabolism have the same outcome in nodulated alfalfa plants—increased nodule number, growth rates, and biomass. To address this, our experimental approach was to compare the two classes of alfalfa plants, *35S‐SPS* and *35S‐GS* transformants, at the molecular, biochemical, and physiological levels under N_2_‐fixing conditions. This would allow us to check for any commonalities or differences in how they might promote growth. Based on our previous study with the *35S‐SPS* alfalfa transformants, we had surmised that the increased growth of those transformants was due to an increase in Suc transport to the nodules (Gebril et al., 2015), resulting in enhanced nodule function, a consequence of an increase in the availability of both energy and C skeletons. The subsequent outcome was an increase in the amount of N transported into the aerial parts, which then contributed to enhanced growth. Based on the literature, the underlying belief with regard to improved performance of plants overexpressing *GS*_*1*_ in a constitutive manner is that the transgene product participates in the reassimilation of photorespiratory ammonia and/or ammonia from nitrate reduction in the leaves and/or the recycling of ammonia produced by the turnover of amino acids (Sengupta‐Gopalan & Ortega, 2015). There is also a study where increased growth of GS_1_ overexpressing poplar trees has been proposed to be due to the role of Gln in the synthesis of indole acetic acid (IAA). A transfer of the amino group from Gln to chorismate produces anthranilate in a reaction catalyzed by anthranilate synthase and anthranilate is involved in the synthesis of IAA (Man, Boriel, El‐Khatib, & Kirby, 2005). While there are a few reports of legume plants transformed with gene constructs to overexpress/downregulate *GS*_*1*_ in either a constitutive or an organ‐specific manner, none of these studies have evoked nodule function to be responsible for increased plant growth (Carvalho et al., 2003; Harrison et al., 2003; Ortega et al., 2004).

Both the *35S‐SPS* and the *35S‐GS* transformants showed an increase in the SPS protein level in the leaves and the nodules. While the increase in SPS levels in the *35S‐SPS* transformants is likely an attribute of the expression of the *SPS* transgene, the increased SPS levels in the leaves and nodules of the *35S‐GS* transformants can only be ascribed to the expression of the endogenous *SPS* genes. Similarly, both classes of transformants showed an increase in the level of GS_1_ protein in the nodules, which in the case of the *35S‐GS* transformants is probably due to the expression of the *GS*_*1*_ transgene. In the case of the *35S‐SPS* transformants, however, the increase in GS_1_ level can only be attributed to the induction of endogenous *GS*_*1*_ genes. Transcript levels for the endogenous *SPSB* gene in the leaves and *SPSA* and *GS1a* genes in the nodules showed a significant increase when compared to the leaves and nodules from control plants. In the leaves, the *35S‐GS* transformants showed the transgene product and there was no increase in the transcript level for either of the endogenous *GS*_*1*_ gene members in both classes of transformants. What is, however, intriguing is that both classes of transformants had the same response with regard to the induction of the endogenous *SPS* and *GS*_*1*_ genes, irrespective of the transgene.

The *35S‐SPS* transformants and the *35S‐GS* transformants showed a 60% and 40% increase in SPS activity in the nodules, respectively. Both sets of transformants also showed an increase in SucS levels in the nodules. We can postulate that the increase in the levels of SPS and SucS enhances the cycle of breakdown and synthesis of Suc (Nguyen‐Quoc & Foyer, 2001), allowing for an increase in the allotment of substrates for N metabolism, the synthesis of starch, cellulose, and signaling molecules. The starch level in the nodules of the two classes of transformants was ~80% higher than in the nodules of control plants. Based on western blot analysis, some of the key enzymes in C and N metabolism were also found to be present in significantly higher levels in the nodules of the *35S‐SPS* transformants and the *35S‐GS* transformants, compared to control, probably an attribute of the increased activation of these genes by a signaling molecule/s.

Both classes of transformants showed an increase in photosynthetic rates as has been reported for other plants overexpressing either *SPS* or *GS*_*1*_ genes (Baxter et al., 2003; Fuentes et al., 2001; Oliveira et al., 2002; Seger et al., 2015). An increase in photosynthetic rates in the *35S‐SPS* transformants can be attributed to an increase in Suc level resulting from the constitutive overexpression of *SPS,* as has been proposed by Baxter et al. (2003). A direct proof for proposing that Suc is the activator for photosynthetic rates was demonstrated by Furbank, Pritchard, and Jenkins (1997) where they showed that photosynthetic rates went up in tobacco leaves when they were fed Suc. Oliveira et al. (2002) attributed the increase in photorespiratory and photosynthetic rates in tobacco plants overexpressing GS_1_ in a constitutive manner to improve reassimilation of photorespiratory ammonia in the mesophyll cells. Thus, in both cases, the end result of increased photosynthetic rates is an increase in Suc levels. We could thus propose that Suc may act as the signal for the induction of *SPSB* in the leaves of both the *35S‐SPS* and *35S‐GS* transformants.

An increase in the level of *SPSA* and *GS1a* transcripts in the nodules of both classes of transformants would imply that the signal regulating *SPSA* and *GS1a* is common between the two sets of plants. It has been suggested that the internal amino acid pools function as a signal to regulate N uptake and assimilation (Miller et al., 2008), and both Gln and Glu are considered important signaling molecules (Forde & Lea, 2007). Carvalho et al. (2003) showed a drop in *GS1a* transcript level in nodules of *M. truncatula* plants grown in the presence of phosphinothricin (PPT; which irreversibly inhibits GS activity) leading to a drop in Gln synthesis. A direct correlation between inhibition of GS activity and *GS1a* transcript level suggests that Gln might have a role in the transcriptional activation of *GS1a*. Both the *35S‐GS* and *35S‐SPS* transformants showed higher levels of *GS1a* transcript, GS_1_ protein, and GS enzyme activity in their nodules compared to controls, thus lending credence to the possibility of Gln being the signal for induction of the endogenous *GS1a* gene. There are, however, no reports of Gln/Glu inducing *SPS* genes.

Besides the induction of *GS1a* and *SPSA* in the nodules of the *35S‐SPS* and *35S‐GS* transformants, the nodules also showed an increase in the levels of AS, neMDH, PEPC, NADH‐GOGAT, and SucS (Figure 9). Carvalho et al. (2003), however, had demonstrated that the genes for enzymes mentioned above were not induced in the nodules of *M. truncatula* plants overexpressing *GS*_*1*_ in a nodule‐specific manner and exhibiting a two‐ to threefold increase in GS activity. This would imply that the inducing signal for the aforementioned genes along with *GS1a* and *SPSA* in the nodules is probably not Gln but some other signaling molecule that is transported from the leaves. These two classes of plants showed a significant increase in Suc concentration in both the leaves and nodules raising the possibility that Suc might be the signal for the induction of *SPSA* and *GS1a* along with the other genes involved in C/N metabolism in the nodules. There are several reports suggesting that besides having a metabolic role, Suc also functions as an effector of gene expression (Ruan, 2012; Smeekens & Hellmann, 2014; Tognetti et al., 2013; Wind et al., 2010). There is evidence in the literature suggesting that Suc plays a role in inducing *GS* genes. Oliveira and Coruzzi (1999) reported that Arabidopsis plants treated with Suc showed an induction of all three of its *GS*_*1*_ genes. Arabidopsis plants expressing antisense gene construct for chloroplastic Fru‐1,6‐bisphosphatase showed not only an increase in Suc content but also an increase in GS_1_ activity, implying that Suc may be inducing *GS*_*1*_ expression (Sahrawy, Avila, Chueca, Cánovas, & Lopez‐Gorgé, 2004). However, there are not too many reports on metabolite‐mediated regulation of *SPS* genes. In some recent work from our laboratory, we have shown that overexpression of *SPS* in a leaf‐specific manner in alfalfa results in an increase in Suc level in both the leaves and nodules and the induction of SPS activity in the nodules (Padhi, 2017). Verma, Upadhyay, Verma, Solomom, and Singh (2011) have demonstrated a positive correlation between Suc levels and SPS activity in sugarcane.

Glutamine synthetase protein accumulation in the nodules of the *35S‐SPS* transformants exceeded that in the *35S‐GS* transformants. While in the *35S‐GS* plants, the GS_1_ protein accumulation in the nodules is accounted for by both the endogenous GS_1_ proteins and transgene product (soybean GS_1_), the *35S‐SPS* transformants, have GS_1_ protein encoded only by the endogenous *GS1a* and *GS1b* genes in their nodules. Since there was no difference in the transcript level for *GS1a* in the nodules between the two sets of plants the question arises as to why the GS_1_ protein level is higher in the nodules of the *35S‐SPS* transformants. Besides regulation at the transcriptional and posttranscriptional levels, GS is also subject to regulation by protein turnover (Ortega et al., 1999; Seabra et al., 2013; Seger et al., 2015; Temple et al., 1994). We could invoke posttranslational regulation to account for this difference in protein levels in the nodules between the two classes of transformants.

Neither one of the two *GS*_*1*_ genes was induced in the leaves of the two classes of transformants. We have proposed that *GS1a* gene is induced by Suc in the nodules of the two classes of transformants. However, *GS1a* was not induced in the leaves even though the Suc concentration was higher in both classes of plants when compared to the control plants. Of the two *GS*_*1*_ genes in alfalfa, the major isoform that is expressed in the nodules is *GS1a* and its expression is minimal in the leaves (Seabra et al., 2013). It is likely that expression of *GS1a* in the nodules requires both nodule factors and Suc imported from the leaves. The increase in the levels of some of the nodule‐enhanced isoforms of key enzymes in C and N metabolism, like neMDH, AS, and SucS, in the nodules of both sets of transformants would imply that the genes encoding these enzymes are also probably regulated by both Suc and nodule factors.

There is evidence that downstream products of N assimilation such as Glu or Gln might serve as the signals of organic N status (Coruzzi & Bush, 2001). Plants have complex regulatory machinery that coordinates the N and C metabolism with nutrient availability, environmental factors, and the demands for growth and development (Nunes‐Nesi, Fernie, & Stitt, 2010). It has been shown that C and N metabolism is coordinated via a mechanism that involves sensing the cellular C and N balance and regulating the transcription of genes involved in several processes such as photosynthesis, respiration, and N assimilation (Gutiérrez et al., 2008; Palenchar, Kouranov, Lejay, & Coruzzi, 2004; Sang et al., 2012. The sensory systems appear to monitor levels of different metabolites, which includes Suc and Gln (Foyer & Noctor, 2006).

While we have tried to implicate Gln or Suc or their derivatives, functioning as signaling molecules to regulate the various genes with a role in C/N metabolism in the nodules, the possibility still exists that both Gln and Suc interact with each other in order to regulate the expression of these genes. Based on feeding cotyledons of *Brassica juncea*, with Suc alone or Suc with a N source (KNO_3_ or NH_4_NO_3_), Goel and Singh (2015) showed that several genes including a gene for cytosolic GS were induced when both C and N were present. Since Gln is the primary product of N assimilation from inorganic N, we could assume that in some cases it is not the inorganic N, rather Gln or Glu, in combination with Suc that act as the signal. Several DNA microarray studies have shown that more than half of the transcriptome is regulated by C, N, and C–N combination (Zheng, 2009). Many enzymes are regulated by both C and N signals (Sang et al., 2012).

An ideal signaling metabolite to coordinate the regulation of N and C metabolism should be in the intersection between these two metabolic pathways. It has long been recognized that the TCA cycle intermediate α‐KG could, in theory, fulfill such a regulatory role and also function as the major C skeleton in N‐assimilatory reactions (Araújo, Martins, Fernie, & Tohge, 2014; Commichau, Forchhammer, & Stulke, 2006). PII proteins are able to sense and integrate signals from central metabolism, in particular α‐KG, an indicator of C/N balance as well as the energy status. PII in bacteria, interacts with other regulatory proteins to modulate GS at the transcriptional and posttranslational level in response to the C/N ratio (Jiang, Mayo, & Ninfa, 2007). It has been speculated that PII in plants could have a role in regulating some steps in C and N metabolism (Chellamuthu et al., 2014; Ferrario‐Méry, Besin, Pichon, Meyer, & Hodges, 2006; Ferrario‐Méry et al., 2005; Forchhammer & Luddecke, 2016; Hsieh, Lam, van de Loo, & Coruzzi, 1998). Besides PII, plants have several known N and C metabolism regulatory pathways like the TOR pathway, Glu receptor, SNF1/AMP‐dependent kinase, and trehalose‐6‐phosphate (Tre‐6P) (Nunes‐Nesi et al., 2010). It is known that the Arabidopsis TOR homologs play roles in nutrient signaling regulating metabolism, growth, and life span (Anderson & Hanson, 2005; Mahfouz, Kim, Delauney, & Verma, 2006; Ren et al., 2012) .

While there are no reports of coordination between *PS* and *GS*_*1*_ expression and of Suc or Gln acting as signaling molecules for their induction, there is evidence in the literature that shows a link between organic acid metabolism and GS activity. Arabidopsis plants expressing a *Dof1* gene from maize showed improved N assimilation and growth under low N conditions. Maize Dof1 is a member of the Dof transcription factors that activates expression of multiple genes associated with organic acid metabolism. Dof1 thus could be a key factor in coordinating gene expression involved in C skeleton production for N assimilation (Yanagisawa, Akiyama, Kisaka, Uchimiya, & Miwa, 2004). The overexpression of *Dof1* increases GS activity and promotes plant growth (Yanagisawa et al., 2004). There is also a report suggesting that a Dof1 transcription factor in pine could have a role in the synthesis of Gln (Rueda‐López, Crespillo, Cánovas, & Ávila, 2008). It would be justifiable to check if Dof1 transcription factor induces the *SPS* genes.

As in the case of the *35S‐SPS* transformants, the *35S‐GS* transformants also exhibited early flowering, increased growth, and biomass, higher photosynthetic rates and increased nodule numbers. Both Suc and Gln have been implicated to play a role in growth and development (Kamada‐Nobusada, Makita, Kojima, & Sakakibara, 2013; Lastdrager, Hanson, & Smeekens, 2014). Gln has been shown to induce the expression of transcription factor genes that are involved in the induction of genes with a role in plant growth (Kan, Chung, Juo, & Hsieh, 2015). It has also been shown that Gln has a role in the synthesis of IAA (Man et al., 2005) and in the regulation of N and cytokinin biosynthesis. Cytokinin regulates a variety of processes in plant growth which includes both root and shoot growth (Matsumoto‐Kitano et al., 2008). Suc is a dominant regulator of growth processes in plants (Lastdrager et al., 2014) and the derivative, Tre‐6P, has been shown to have a role in plant growth (Yadav et al., 2014). Suc is known to induce auxin levels (Lilley, Gee, Sairanen, Ljung, & Nembhauser, 2012; Sairanen et al., 2012), and also induces auxin transport and signal transduction in Arabidopsis (Stokes, Chattopadhyay, Wilkins, Nambara, & Campbell, 2013). In more recent studies, it has been shown that Suc rather than auxin is possibly responsible for apical dominance (Mason, Ross, Babst, Wienclaw, & Beveridge, 2014). Early flowering in both sets of plants, as proposed by Gebril et al. (2015), could be in response to increased Suc in the phloem (Corbesier, Bernier, & Périlleux, 2002).

In this study, it appears that the *35S‐SPS* transformants exhibit higher growth rates than the *35S‐GS* transformants during the early stages following inoculation, which could be attributed to increased nodule number in the *35S‐SPS* transformants compared to the *35S‐GS* transformants and control. That raises the question of why the *35S‐SPS* transformants have more nodules than the *35S‐GS* transformants and control plants. The regulatory pathway that controls the number of nodules in the roots has been shown to be mediated by signaling molecules and phytohormones (Ferguson et al., 2010). The same signaling molecules also interact or crosstalk with sugar pathways in the regulation of many other pathways (Engels, Kirby, & White, 2011; Hammond & White, 2008). Nodule initiation may be influenced by the Suc level and it is likely that the *35S‐SPS* transformants, due to the constitutive expression of the *SPS* transgene export more Suc to the site of nodule initiation via their root system when compared to the *35S‐GS* transformants and the control plants. The overexpression of *GmNMHC5,* a MADS box containing transcription factor, in transgenic soybean, significantly promoted lateral root development and nodule building. *GmNMHC5* is upregulated by exogenous Suc. These results indicate that GmNMHC5 can sense the Suc signal and plays significant roles in lateral root development and nodule building (Liu et al., 2015).

The phenomenon of endogenous *GS*_*1*_ and *SPS* genes being upregulated in plants overexpressing either *SPS* or *GS* appears to be unique for alfalfa but then there are no reports of this kind of analysis in any other leguminous plant. We have shown that tobacco transformants containing the *35S‐GS* gene construct, while showing an increase in GS_1_ level, did not show an increase in SPS level, SPS enzyme activity or Suc content in the leaves when compared to the control plants. Similarly, the *35S‐SPS* tobacco transformants did not show an increase in GS protein levels or GS enzyme activity (Seger et al., 2015). Leguminous plants could be unique in that they have nodules, the site of N_2_‐fixation and ammonia assimilation. The root nodules act both as the C sink and as the N source and the leaves act as the C source and N sink requiring that there be close cross talk between C and N metabolic pathways and between the nodules and leaves. This would require fine‐tuning in the expression of the endogenous *GS* and *SPS* genes.

One of the hallmarks of good forage quality is low fiber content and this has been accomplished in alfalfa by genetic manipulation of the lignin pathway (Guo et al., 2001; Shadle et al., 2007). It is, however, important to note that improvement in forage quality cannot be at the cost of biomass. Gallego‐Giraldo et al. (2016) have shown that significant increase in forage quality and biomass can be increased by downregulating the expression of a family of WRKY transcription factors that act as the repressor of secondary cell wall formation. We have shown here that the *35S‐SPS* and *35S‐GS* plants, besides showing increased growth and biomass, also have superior forage quality—relatively higher protein content and/or lower fiber content. The increase in protein content in the aerial parts of the plant can be attributed to an increase in N assimilation in the nodules resulting in increased availability of amino acids for protein synthesis in the aerial parts of the plant. The only explanation that we have to offer for a decrease in fiber content is that secondary cell wall synthesis may not be able to keep up with the faster growth rates in these two classes of transformants. A point to note here is that the *35S‐SPS* plants have 10% lower fiber content compared to the *35S‐GS* transformants. This could be accounted for by the expression of the *GS*_*1*_ transgene in the vasculature of the *35S‐GS* transformants (Seger et al., 2009), which is the major site for lignin synthesis. GS_1_ plays a role in the reassimilation of ammonia released from phenylalanine ammonia lyase during lignin production and allows the maintenance of high rates of lignification without affecting the N economy (Cánovas, Avila, Canton, Canas, & de la Torre, 2007). The higher lignin content in the *35S‐GS* transformants could account for the higher biomass in this class of transformants compared to the *35S‐SPS* transformants. Future studies will focus on performing detailed analysis of the sugars, polysaccharides and lignin in these two classes of transformants.

## 5 CONCLUSIONS

By comparing the two classes of transformants, *35S‐SPS* and *35S‐GS*, we have shown that the biochemical basis for improved growth is the same, even though SPS and GS are key enzymes in C and N metabolism, respectively. Some of the growth attributes in the *35S‐GS* transformants could be a consequence of improved reassimilation of photorespiratory ammonia. In both sets of plants, improvement in plant performance can be attributed to enhanced sink strength of the nodules, which could be the outcome of increased synthesis of Suc in the source leaves (Kaschuk, Hungria, Leffelaar, Giller, & Kuyper, 2010). It would thus appear that a common signaling mechanism exists in both sets of plants that regulate the expression of key genes with a role in nodule function. We could also entertain the possibility that Suc activates a transcription factor/s which in turn regulates all the key genes needed for nodule function. It is important to point out that even though the two genes, *SPS* and *GS*_*1*_, used for overexpression in this study, target two different metabolic pathways, the two sets of plants undergo molecular changes such that they end up being similar with regard to the levels of SPS and GS_1_ proteins in the nodules.

While both Suc and Gln (or their derivatives) have been implicated to function as signaling molecules that induce gene expression, the data presented here is more supportive of Suc being the signaling molecule. Taken together, the results demonstrate the close interaction and interdependence between C and N metabolism. Furthermore, this study reaffirms the utility of the nodulated legume plant to study C/N interaction and the cross talk between the source and sink for C and N. Experiments are in progress to manipulate expression of *SPS* and *GS* in an organ‐specific manner.

## CONFLICTS OF INTEREST

The authors declare no conflict of interest associated with the work described in this manuscript.

## AUTHOR CONTRIBUTIONS

C.S.G. and J.L.O. supervised the experiments; H.K. and A.P. performed the experiments; K.A. and S.G. provided technical assistance; C.S.G. conceived the project and wrote the article with contributions of H.K, A.P., and J.L.O.
