# Supplementary material for: Comparison of alfalfa plants overexpressing glutamine synthetase with those overexpressing sucrose phosphate synthase demonstrates a signaling mechanism integrating carbon and nitrogen metabolism between the leaves and nodules
Source: Plant Direct. 2019 Jan 31;3(1):e00115. doi: 10.1002/pld3.115 (PMC6508842; doi:10.1002/pld3.115)
Supplement: Supplementary file 1 [file PLD3-3-e00115-s001.pdf]

**College of Agricultural, Consumer & Environmental Sciences**
Plant & Environmental Sciences
MSC 3Q
New Mexico State University
P.O. Box 30003
Las Cruces, NM 88003-8003
Phone: (575) 646-3405

Dr. Fortes
Academic Editor
Plant Direct

December 19, 2018

Dear Dr. Fortes

I want to start by thanking you and the reviewers for all your comments and critiques. We have incorporated most of the suggested changes and I will list all the changes made in response to the comments made by the reviewers. In a few instances, we did not agree with the reviewers and did not make the suggested changes. We have given our reasoning behind that. We feel the manuscript has improved immensely with all the changes that we have made in response to all the critiques. In the following section, I will respond to each comment made by the two reviewers.

**Response to Reviewers' comments**

**REVIEWER #1**

**Comment**: There is a need to underline what is especially new, added-value of this MS in connection to earlier work of this group
**Response**: Legumes are unique in that they have nodules, which function as a C sink and N source while the leaves function as N sinks and C source, thus making it an ideal system for studying C/N interaction and the cross talk between the source and sink for C and N. To determine if there is any inter/co- dependence between C and N metabolic pathways between the leaves and nodules, this study was undertaken to compare $GS_1$ overexpressing alfalfa plants (*35S-GS*) with the *SPS* overexpressing alfalfa plants (*35S-SPS*). Moreover, *35S-GS* alfalfa transformants have not previously been characterized by either our lab or any other lab.

**Comment:** Line 144 – please rephrase
**Response:** Have removed that phrase

**Comment**: Line 580 – connect or connection
**Response**: The sentence has been reworded

**Comment:** Overall Discussion section should be shortened

**Response:** The 'Discussion' section has been shortened by removing the text that is not directly relevant to the main theme of the paper

**Comment:** Conclusions are far too speculative.
**Response:** Have made it less speculative.

**Comment:** More figures instead of some tables
**Response:** converted 5 of the 6 tables into figures

**REVIEWER #2**

**Comment:** The introduction is quite poor -----
**Response:** We have modified the 'Introduction' by incorporating some of the new information on **N and C metabolism as it occurs in illuminated leaves**. However, with regards to the root nodules, it is widely accepted that C and energy in the nodules is derived from the sucrose that is imported into the nodules from the leaves. To the best of our knowledge, there have been no studies done to look at the flux. Moreover, the focus of the paper is to examine the relationship between leaves and nodules with regards to C and N. Since perturbing either one of the two metabolic pathways by overexpressing either *GS* or *SPS*, has the same outcome at both the molecular and biochemical level, we feel justified in saying that **there could** be a common signaling mechanism in regulating the expression of genes involved in nodule function, in both classes of transformants.

**Comment:** Lines 62 – 66. This is a very old picture ------
**Response:** As mentioned above, the work from Tcherkez lab, has to do with the interactions between dark and light metabolisms in the leaves and the origin of C in the glutamate made in the leaves. The focus of our work, however, has to do with the interaction between C and N metabolism in the leaves and nodules.

**Comment**: Lines 93 – 96. Are you sure this C comes from Suc?
**Response:** The reviewer is questioning if the source of C for Glu/Gln is sucrose or citrate (and other organic acids). No flux studies have been done in the root nodules of any legumes and as such we cannot address this question. Moreover, we are only stating that the source of C and energy for both $N_2$-fixation and ammonia assimilation in the nodules is sucrose transported from the leaves. Molecular work from our lab has shown that overexpression of *SPS* in a leaf-specific manner leads to an increase not only in sucrose content but also in GS activity in the nodules. Downregulation of sucrose synthase in a nodule-specific manner shuts down $N_2$-fixation in the nodules, implying that sucrose breakdown is needed for nodule function. We have, however, referred to the papers from Tcherkez lab, in the 'Introduction' section.

I hope the manuscript in its present form will be reviewed favorably.

Thank you.

Sincerely yours

Champa Sengupta-Gopalan, Professor
E-mail: csgopala@nmsu.edu
